# Stitching the synapse: Cross-linking mass spectrometry into resolving synaptic protein interactions

**DOI:** 10.1126/sciadv.aax5783

**Published:** 2020-02-19

**Authors:** M. A. Gonzalez-Lozano, F. Koopmans, P. F. Sullivan, J. Protze, G. Krause, M. Verhage, K. W. Li, F. Liu, A. B. Smit

**Affiliations:** 1 Department of Molecular and Cellular Neurobiology, Center for Neurogenomics and Cognitive Research, Amsterdam Neuroscience, Vrije Universiteit Amsterdam, Amsterdam, Netherlands.; 2 Department of Medical Epidemiology and Biostatistics, Karolinska Institute, Stockholm, Sweden.; 3 Departments of Genetics and Psychiatry, University of North Carolina, Chapel Hill, NC, USA.; 4 Department of Structural Biology, Leibniz-Forschungsinstitut für Molekulare Pharmakologie (FMP), Berlin, Germany.; 5 Department of Functional Genomics, Center for Neurogenomics and Cognitive Research, Amsterdam Neuroscience, Vrije Universiteit Amsterdam, Amsterdam, Netherlands.; 6 Department of Chemical Biology, Leibniz-Forschungsinstitut für Molekulare Pharmakologie (FMP), Berlin, Germany.; 7 Biomolecular Mass Spectrometry and Proteomics, Bijvoet Center for Biomolecular Research, and Utrecht Institute for Pharmaceutical Sciences, Utrecht University, Utrecht, Netherlands.

## Abstract

Synaptic transmission is the predominant form of communication in the brain. It requires functionally specialized molecular machineries constituted by thousands of interacting synaptic proteins. Here, we made use of recent advances in cross-linking mass spectrometry (XL-MS) in combination with biochemical and computational approaches to reveal the architecture and assembly of synaptic protein complexes from mouse brain hippocampus and cerebellum. We obtained 11,999 unique lysine-lysine cross-links, comprising connections within and between 2362 proteins. This extensive collection was the basis to identify novel protein partners, to model protein conformational dynamics, and to delineate within and between protein interactions of main synaptic constituents, such as Camk2, the AMPA-type glutamate receptor, and associated proteins. Using XL-MS, we generated a protein interaction resource that we made easily accessible via a web-based platform (http://xlink.cncr.nl) to provide new entries into exploration of all protein interactions identified.

## Teaser

Cross-linking mass spectrometry provides a resource for the discovery of novel protein interactions of synaptic protein complexes.

## INTRODUCTION

Synapses are fundamental signaling units of the brain orchestrating fast information transfer between neurons, as well as between neurons and peripheral tissues. Specific synaptic protein complexes are crucial to information processing and storage (*1*), whereas their disease-related disruption, known as synaptopathies, are prevalent causes of brain disorders, such as autism, epilepsy, intellectual disability, and schizophrenia (*2*). Synapses are semiautonomous organelles with the ability to execute general cellular and metabolic processes, e.g., de novo protein synthesis, protein turnover by targeted degradation, and energy production independently of the cell soma. Apart from these, synaptic transmission and plasticity require specialized molecular machineries composed of specific proteins engaged in transient or stable protein-protein interactions (PPIs) acting at the pre- and/or postsynaptic compartments (*3*). Typically, in the presynapse, neurotransmitter-loaded synaptic vesicles are docked at the active zone by scaffold proteins for the subsequent membrane fusion driven by calcium-dependent conformational changes of calcium sensors and SNARE (soluble *N*-ethylmaleimide–sensitive factor attachment protein receptor) protein interactions. At the postsynapse, the postsynaptic density hosts diverse functions in neurotransmission, such as anchoring ionotropic and metabotropic glutamate receptors, physically connecting and aligning pre- and postsynaptic elements by transsynaptic cell adhesion molecules, recruiting intracellular signaling effectors, e.g., Ca^2+^/calmodulin-dependent protein kinase II (CaMKII/Camk2) or G proteins, and linking these components to the actin cytoskeleton (*4*). Proteomic approaches have identified over 2000 different proteins at the synapse (*5*, *6*), comprising approximately 10% of protein-coding genes in mammalian genomes. Because of this complexity, the description of synaptic architecture and its extended protein interaction network is still a major challenge.

The function and organization of synapses critically depend on protein interaction and structure, typically resolved by affinity purification–based proteomics and high-resolution x-ray crystallography or cryo–electron microscopy technologies, respectively. Complementary to these approaches, chemical cross-linking combined with mass spectrometry (XL-MS) offers the advantage of capturing both native protein structures and interactions by cross-linking reagents in a physiologically relevant subcellular context (*7*). The spatial close proximity of cross-linked sites can be used to reveal protein binding interfaces, novel partnerships, as well as protein conformations and dynamics. With recent technical advances, global XL-MS analysis of untargeted protein complexes and structures has become feasible (*8*).

In this study, we applied XL-MS to probe the complex architecture of the synaptic compartment. We identified 11,999 unique lysine-lysine cross-links from 2362 different proteins, representing one of the largest cross-linking datasets (available online at http://xlink.cncr.nl). Using various biochemical and computational approaches, we validated the fidelity of our procedure and investigated protein interactions and dynamics for key synaptic proteins, such as Camk2 and the AMPA receptor (AMPAR). This extensive resource provides a novel perspective on protein structures, assemblies, and interactions of the synaptic proteome.

## RESULTS

### XL-MS analysis of the synapse

**Fig. 1:**
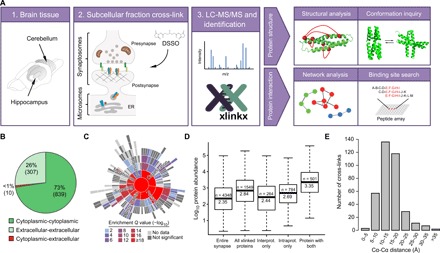
Overview of XL-MS workflow and results. (**A**) Schematic workflow of XL-MS and its applications. ER, endoplasmic reticulum. (**B**) Pie chart showing the number of cross-links identified between cytoplasmic and extracellular regions of proteins (dataset 1). (**C**) Sunburst plot showing the annotation in synaptic functions of the cross-linked proteins identified [biological processes SynGO terms (*11*)]. Inner rings are parent terms of more specific child terms in the outer rings, color coded according to enrichment *Q* value. Notably, a wide and significant coverage of synapse-specific proteins was found distributed across both pre- and postsynapse functions. (**D**) Boxplot showing the distribution of protein abundances in different categories: Entire synapse, all proteins identified in standard proteomic analysis; All cross-linked proteins, proteins identified in our XL-MS dataset 1; Interprot. only, proteins involved in only interprotein cross-links; Intraprot. only, proteins involved in only intraprotein cross-links; and Protein with both, proteins involved in both intra- and interprotein cross-links. The median abundance for cross-linked proteins is three times higher than the synaptic proteome and 10 times higher for proteins involving both intra- and interprotein links, showing the influence of protein abundance in the detection of cross-links. Number of proteins and the median abundance are indicated in each box. Protein abundance data were obtained from (*12*). (**E**) Distribution of Cα-Cα distances of cross-links from eight selected protein complex structures (fig. S2, A to C).

We applied proteome-wide XL-MS using the MS-cleavable cross-linker DSSO (disuccinimidyl sulfoxide) to reveal the architecture and assembly of synaptic protein complexes (Fig. 1A). To extensively cover the synaptic proteome, we analyzed two subcellular fractions, synaptosomes (widely synapse enriched) and microsomes (membrane enriched for proteins involved in assembly and trafficking), from mouse hippocampus and cerebellum. The isolated subcellular fractions were immediately cross-linked with the DSSO cross-linker to best preserve the protein structures and interactions. Cross-linked proteins were digested and analyzed by mass spectrometry, as previously described (*7*, *9*). Data analysis was performed using XlinkX v2.0 (*10*), imposing a 2% false discovery rate (FDR). This dataset comprised 7135 unique Lys-Lys cross-links (dataset 1 in table S1A), including 1552 interprotein cross-links (1036 different protein pairs) and 5583 intraprotein cross-links (within 1472 proteins). We created an interactive web tool to facilitate the inspection of all the cross-links identified (http://xlink.cncr.nl), including the number of samples in which each cross-link was found and the associated FDR (also available in table S1A), which allows the user to evaluate the reliability of specific cross-links. We also included filtering options that enable advanced visualization of experiment subsets, retrieving the origin of the cross-links, the FDR of the identification, and overlay with previously reported protein interactions.

To assess whether XL-MS correctly captured the spatial localization of the synaptic proteins, we investigated the existence of aberrant cross-links between intra- and extracellular regions of proteins (Fig. 1B). We retrieved all the topological information available in UniProt for the cross-linked proteins in the dataset. From the 1156 cross-links mapped to known cytoplasmic or extracellular regions, only 10 cross-links (0.87%) were found between the cytoplasmic and extracellular regions. This observation suggests the structurally intactness of the synaptosomes in the preparations used to generate the cross-linking dataset.

To reveal the coverage of synapse-specific proteins in the cross-linking repertoire, we interrogated the recently established synaptic Gene Ontology (GO) database SynGO (*11*), which includes 1112 synaptic proteins based on expert-curated literature evidence. From the cross-linked proteins identified, 512 mapped to SynGO annotated genes (table S1B). Fifty-seven biological processes terms were significantly enriched across all main synaptic functions (such as presynapse, postsynapse, signaling, organization, metabolism, and transport; Fig. 1C) and 32 cellular component terms (fig. S1B), when compared with all brain expressed genes as background. This demonstrates the coverage of a wide spectrum of synaptic proteins. Moreover, a range of cross-linked proteins identified was found beyond the currently annotated SynGO proteins, including general cellular and metabolic proteins known to reside in the synapse.

The depth and sensitivity of the XL-MS approach were interrogated by comparing the abundances of proteins cross-linked over the entire synaptic proteome. Protein abundance data were obtained from our previous study using label-free liquid chromatography–MS (LC-MS) quantification on equivalent preparations (*12*). The median abundance of the proteins identified by XL-MS was three times higher than the median abundance of the synaptic proteome (Fig. 1D). This difference was more pronounced (10 times higher) when comparing proteins involving both intra- and interprotein links. Only a weak correlation (Pearson *R*^2^ = 0.23) was found for the number of cross-linked lysines and the protein abundance corrected for the total number of lysine residues (fig. S1, B and C). No correlation was found between the number of cross-linked lysines and the total number of lysines of each protein (fig. S1D) for protein abundance and the total number of lysines (only a minor effect depending on the sequence length; fig. S1, E to G). Together, these data indicated that high-abundance proteins were preferentially identified, while less abundant proteins were detected more sporadically.

The formation of cross-links is bound to the spatial close proximity between two lysine residues embedded in the three-dimensional structure of protein complexes. Thus, the confidence of XL-MS datasets can be interrogated by inspecting the spatial distances of the cross-linked pairs on previously reported high-resolution structures. We matched the cross-link data with eight well-established synaptic, mitochondrial, and ribosomal protein complexes (fig. S2, A to C). More than 99% of the unique lysine-to-lysine cross-links within the structures (363 in total) fell below the distance limit imposed by the DSSO cross-linker (23.4 + 10 Å, considering in-solution flexibility; Fig. 1E) (*13*), which supports the fidelity of the cross-linking assay. Furthermore, since protein structures are often elucidated in nonphysiological experimental conditions, cross-linking represents a complementary approach to validate and further explore native protein structures in their fractionated subcellular context.

### Capturing the structural dynamics and flexibility of Camk2 kinase domains

**Fig. 2:**
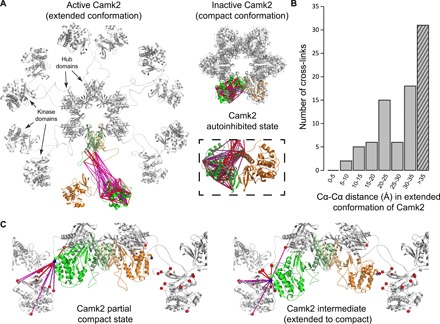
Conformational modeling of Camk2 kinase domains. (**A**) Cross-link mapping on the extended (PDB 5U6Y), the compact (PDB 3SOA), and the autoinhibited conformation of Camk2 (shown as autoinhibited dimer of kinase domains, PDB 2BDW and fig. S2D). Two highlighted monomers are shown in green and orange, respectively. For clarity, cross-links are mapped onto the monomer in green only. (**B**) Distribution of Cα-Cα distances of cross-links from Camk2 mapped onto the extended state (PDB 5U6Y). The hatched pattern indicates cross-links exceeding DSSO maximal distance restraint. (**C**) Illustration of domain flexibility and alternative conformational states of Camk2. In all conformational states, six monomers of Camk2 (half of the complex) are presented. Proposed models of a partial compact state (based on PDB 5U6Y and 3SOA) and a transient intermediate state between the extended and compact state (snapshot of morph shown in movie S1) are depicted. Cross-links between K258 (a major cross-linking site in the kinase domain, shown as blue sphere) and other lysines (red spheres) are highlighted. Cross-links are represented as the Euclidean distance between Cα atoms of the cross-linked residues, indicated in red (if below DSSO maximal distance restraint) or in magenta (if exceeding DSSO maximal distance restraint).

In combination with structure modeling and dynamic simulation approaches, XL-MS can facilitate the investigation of different conformational states and in-solution flexibility of unstructured regions in their native fractionated subcellular conditions. Here, we focused on CaMKII/Camk2, an essential synaptic protein kinase that is crucially involved in the signaling cascade required for synaptic plasticity underlying learning and memory. Monomeric Camk2 is composed of an N-terminal kinase domain, a regulatory region, a highly flexible linker region, and a C-terminal association (hub) domain, which further assembles into a large 12-mer holoenzyme via the C-terminal association domains. It undergoes large activity-dependent conformational movements, i.e., active or inactive in extended or compact conformation, respectively. The observed Camk2a and Camk2b cross-links were mapped onto three different Camk2 structures (Fig. 2A), including a full-length compact state [Protein Data Bank (PDB) 3SOA], a full-length extended state (PDB 5U6Y), and a kinase domain–only structure representing the autoinhibitory dimerization of the kinase domain (PDB 2BDW). Furthermore, the kinase domain–only structure was combined with the extended structure (PDB 5U6Y) to generate a full-length model of the autoinhibited state (fig. S2D), allowing the exploration of cross-links between the kinase domain dimer and the other regions of the protein. In contrast with the previous Camk2 protein structures, our data show that 35% of the cross-links mapped onto the extended state of Camk2 exceeded the cross-linker maximum distance restraint (Fig. 2B), supporting the existence of large conformational changes. We then focused on K258-centered cross-links because it is a major cross-linking site at the kinase domain. We observed 11 cross-links of K258 that did not comply with any of the three structures examined (Fig. 2A), including cross-links between the kinase domain and the rigid central hub, as well as in between monomeric kinase domains. This is in agreement with the high flexibility of the kinase domain of Camk2 via the linker region. Furthermore, based on a number of cross-links that cannot be explained purely by domain flexibility, we proposed that two neighboring kinase domains may be arranged as one in compact and the other in extended state within the same structure (partial compact state; Fig. 2C). We summarized the collection of possible contacts between kinase domains by inferring the movement between states using analysis of dynamic regions of Camk2 and an interpolated trajectory between states (transient intermediate state; Fig. 2C and movie S1). Collectively, our cross-linking approach captured highly dynamic and extremely large movements between the kinase domains and the rigid central hub of Camk2 and suggested multiple contacting possibilities between kinase domains upon activation and binding to protein partners.

### XL-based network for novel PPI discovery

**Fig. 3:**
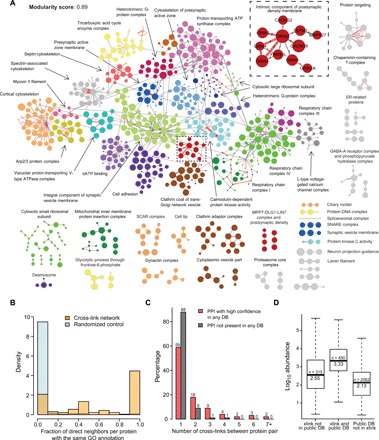
Characterization of XL-based protein interaction network. (**A**) Core component and separate modules of the combined network (dataset 1). Nodes represent proteins, and edges show the interactions identified by XL-MS. Nodes are color coded based on functional clusters generated by unsupervised edge-betweenness clustering and annotated by GO enrichment analysis. Disconnected modules were grouped using DAVID Gene Functional Classification, and each group was subsequently annotated. Edges in red indicate protein-protein interactions (PPIs) reported in the literature (high confidence in at least one database of STRING, InWEB, and BioGRID). Sizes of the nodes are proportional to the protein abundances (log_2_ scale). Widths of the edges are proportional to the number of unique Lys-Lys cross-links. Insert represents the enlarged cluster annotated as intrinsic component of postsynaptic density membrane, with the edges labeled according to the number of samples in which the PPI was identified. Network representations of individual experiments are provided in figs. S3 to S6. dATP, 2′-deoxyadenosine 5′-triphosphate. (**B**) Distribution of the number of cross-linked proteins pairs annotated within the same GO group in the XL-based network (labeled in orange) and a randomly rewired network (100 iterations, labeled in light blue). (**C**) Distribution of the number of cross-links found between protein pairs present in at least one database (DB) with high confidence (labeled in red) or not present in any of the three databases (i.e., STRING, InWEB, and BioGRID; labeled in black). (**D**) Boxplot showing the distribution of protein abundances in different categories: xlink, cross-linked proteins identified in this study; and public DB, protein interactions present in at least one public database with high confidence (i.e., STRING, InWEB, and BioGRID). The number of proteins and median abundance are indicated in each box. Protein abundance data were obtained from (*12*).

The identification of 1036 protein pairs cross-linked enabled us to construct an XL-based protein-protein interaction map of the synapse. The PPI network of individual subcellular fractions and brain areas are shown in figs. S3 to S6, and the combined network is shown in Fig. 3A (from dataset 1). The core component of the combined network comprised 577 interconnected proteins, with another 218 proteins forming separate modules with at least three proteins connected (Fig. 3A and fig. S7). The number of samples in which each PPI was identified is indicated in fig. S7. The degree distribution of the combined network follows a power law log-log fit (fig. S8, A and B), representing a typical scale-free network topology that complies with previously reported characteristics of protein interactomes (*14*). The core component showed a strong modular organization (modularity score 0.89), with a total of 25 protein clusters defined by unsupervised edge-betweenness clustering (Fig. 3A and table S2). The established clusters were significantly enriched for specific GO terms (Fig. 3A), including major synaptic subcompartments and protein assemblies such as “intrinsic component of postsynaptic density membrane,” “L-type voltage-gated calcium channel complex,” “cell adhesion,” and “integral component of synaptic vesicle membrane” (table S2). Next, we tested the proximity of the proteins within each cellular component term in the entire network by measuring the fraction of cross-linked protein pairs of which the proteins were annotated with the same GO term, in comparison to a randomized rewired control (Fig. 3B). We found that the proteins in the network were significantly more connected to other proteins from the same cellular component (two-sample Kolmogorov-Smirnov test, *P* = 2.2 × 10^−16^). Similarly, the path distance between the annotated proteins for each GO term was shorter compared with the nonannotated proteins for the same term (fig. S8C). For each individual network, proteins were also found highly connected to proteins from the same cluster (two-sample Kolmogorov-Smirnov test, *P* < 10^−15^; figs. S3 to S6B). Thus, these network topology analyses argue for the capability of XL-MS in capturing authentic protein connectivity.

To compare the XL-based PPIs to previously reported protein interactions, we overlaid the cross-linked protein pairs to the experimentally defined interactions retrieved from the public PPI databases STRING (*15*), InWEB (*16*), and BioGRID (*17*), which together represent a curated collection from 11 original sources (e.g., IntAct and BIND). As a result, 51% of the total protein pairs identified (531 protein pairs) were present in the public databases, suggesting that a large part of the cross-links potentially represent novel protein interactions (fig. S8D). To retrieve the most reliable PPIs from the databases, we classified each PPI in different confidence levels (low, medium, and high), depending on the data curation method applied in the database. We found that 39% of the cross-linked protein pairs represented PPIs with high confidence from at least one database. We observed that these 39% of cross-linked protein pairs contained a higher number of cross-links compared with the cross-linked protein pairs not present in the databases (Fig. 3C). Furthermore, high-confidence protein interactions present in both XL-MS and PPI databases were represented by proteins with more than six times higher median abundance than those absent in the databases (Fig. 3D). Similar results were obtained for the individual networks (figs. S3 to S6). These observations imply that proteins with higher abundances are more likely to be detected by both XL-MS and other experimental approaches included in public PPI databases.

### Validating PPI sites of SNARE proteins

XL-MS provides information at the sequence level that can be used to reveal protein-protein binding interfaces. To explore this, we first investigated the distribution of cross-linked lysines regarding human protein interaction interfaces as predicted by Interactome INSIDER (*18*). We mapped the cross-linked lysines derived from our mouse data to the human proteome, generating 1512 lysine positions with a confident mouse-human mapping (table S3). A significant enrichment of interprotein cross-linked lysines located within the protein interaction interfaces was found (47%, Fisher’s exact test <0.00001), while intraprotein cross-linked lysines were not enriched (26%, Fisher’s exact test 0.79; fig. S9A). Thus, interprotein cross-linked sites seem to frequently occur within protein-protein interaction regions.

**Fig. 4:**
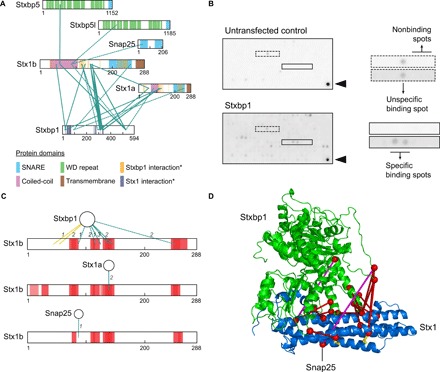
Peptide array analysis of selected cross-linked proteins. (**A**) Cross-link maps of selected SNARE proteins (dataset 1). Protein domains are depicted based on UniProt database and previous publications [*, from (*31*)]. Identified cross-links located in unique and shared peptides of Stx1a and Stx1b are represented as solid and dotted lines, respectively. (**B**) Results of Stxbp1 and control peptide array experiments. Examples of specific, nonspecific, and no binding signals are depicted. Arrowheads indicate antibody positive controls. Quantification and peptide sequences (two independent and two technical replicates) are described in fig. S9B and table S4. (**C**) Comparison of binding regions determined by peptide array (boxes colored in red) and XL-MS (edges). The number of samples in which the cross-links were identified is indicated on the edges (dataset 1). For peptide array assays, full-length proteins are shown as circles, and the protein used to generate peptide sequences in the arrays (Stx1b) is represented as sequence bars. Yellow edges match arrows with the same color in (D). (**D**) Cross-link mapping of Stxbp1 interactions. Stxbp1 (green) and Stx1 (blue) cross-linked sites were mapped onto the Stxbp1-Stx1 complex structure (PDB 3C98; Stx1 in closed conformation). Cross-links are indicated in red (if below DSSO maximal distance restraint) or in magenta (if exceeding DSSO maximal distance restraint). Yellow sticks show the two lysine residues of Stx1 (Lys45 and Lys55) found outside of the binding region with Stxbp1 as defined by the peptide array. Black edge indicates cross-linked site of Snap25.

Second, we performed peptide array interaction assays to independently assess whether cross-linked lysine pairs are involved in experimentally verifiable interaction sites (Fig. 4A). Five presynaptic SNARE proteins, namely, Stxbp1, Snap25, Stx1a, Stxbp5, and Stxbp5l, were expressed in human embryonic kidney (HEK) 293 cells, extracted, and incubated with a peptide array presenting peptides with tile-wise overlapping amino acid sequence of the entire Stx1b (table S4). The interaction between protein and peptide was detected by immunoblotting in two independent replicates and two technical replicates (Fig. 4B and fig. S9B). Fluorescent signals were quantified, normalized, and the nonspecific binding was subtracted (fig. S9B). The binding sites of Stxbp5 and Stxbp5l with Stx1b could not be validated due to the low signals observed. Only proteins binding to at least two peptides with overlapping sequence were considered true interactions and shown in interaction maps (Fig. 4C). Notably, the majority of the binding regions detected by peptide array were found close to the cross-linking sites (Fig. 4C). Using XL-MS and peptide array, we confirmed previously reported protein interactions and their binding sites, including Stx1 with Stxbp1 and Snap25 (Fig. 4C).

The cross-inked lysine residues connecting Stxbp1, Stx1a, Stx1b, and Snap25 were mapped onto the three-dimensional structure of the Stxbp1-Stx1 interaction in closed conformation (PDB 3C98). The missing loops from the crystal structures of Stxbp1 (residues 506 to 531) and Stx1 (residues 10 to 26) were modeled with I-TASSER (Fig. 4D). The majority of cross-links detected between Stxbp1 and Stx1 were located at the proximity between the two proteins and were within the maximal Cα-Cα distance restraint (15 cross-links were below 27 Å). Two long-distance cross-links were found between Stxbp1 and Stx1: one within the Stxbp1 loop region modeled by I-TASSER and the other within the Habc domain of Stx1. The first cross-link is likely due to the extremely high flexibility of the Stxbp1 loop, while the second may correspond to an alternative conformation of Stx1 [e.g., open conformation as previously proposed (*19*)] as opposed to the closed conformation used here. In addition, two cross-linked sites of Stx1b (Lys45 and Lys55) were found approximately 20 amino acids away from the binding site between Stxbp1 and Stx1b as defined by the peptide array (Fig. 4D, yellow edges). In the three-dimensional structure, these lysines are facing the opposite side of the binding interface of the Stx1-Stxbp1 interaction (Fig. 4D), suggesting that they may not directly contribute to this interaction but still can be cross-linked due to their close vicinity to the binding site. Together, using XL-MS and independent approaches, we were able to validate the binding sites of several protein-protein interactions.

### Modeling the AMPAR auxiliary protein complex

**Fig. 5:**
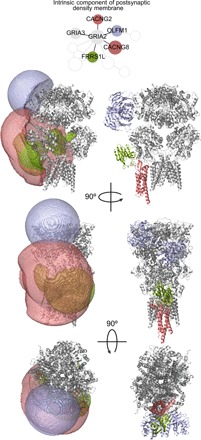
Analysis of auxiliary protein interactors of the AMPAR. Interaction space models (semitransparent volume; left) and docking models (right) of the interactions between AMPAR (PDB 5IDE) and its known interactors Olfm1 (PDB 5AMO), Frrs1l DOMON domain (modeled based on PDB 4ZEL), and Cacng2/8 (PDB 5VOT) were generated on the basis of the XL-MS data. Interaction space models were calculated using the DisVis Webserver, and docking models were generated with HADDOCK2.2. Cacng2 was structurally aligned from PDB 5VOT.

From the cross-linked proteins identified, we investigated the AMPAR in more detail. The AMPAR is a key ligand-gated ion channel that mediates the fast neurotransmission and plasticity in the postsynapse. Its activity, function, and properties are tightly regulated by the interaction with several auxiliary proteins. Here, we combined structural modeling and interaction site investigation on the GO cluster “intrinsic component of the postsynaptic density” to provide further insights into protein interaction and assembly of the AMPAR (Fig. 5A). This cluster consists of 10 proteins, including the AMPAR complex (Gria1–4) as well as the previously established AMPAR interactors Frrs1l, Cacng2, Cacng8, and Olfm1 (*20*). The use of synaptosomes and microsome fractions from hippocampus and cerebellum showed combinations of AMPAR subunits and auxiliary proteins known to be enriched in these specific brain regions and subcompartments, such as Cacng8 in the hippocampus, Cacng2 (*21*) and Gria4 (*22*) in the cerebellum, and Frrs1l (*23*) in the endoplasmic reticulum (ER). On the basis of the identified cross-links, we performed interaction space analysis using the DisVis Webserver and generated docking models using HADDOCK2.2 for the interactions between the AMPAR (PDB 5IDE; showing the physiologically relevant Gria2/3 composition) and its known binding partners Olfm1 (PDB 5AMO), Frrs1l, Cacng2, and Cacng8 (Fig. 5A). The dopamine β-monooxygenase N-terminal (DOMON) domain structure of Frss1l was modeled based on sequence homology using the available structure with highest sequence coverage (PDB 4ZEL) and the cross-links involved in both Cacng2 and Cacng8 were mapped onto the structure of Cacng2 (PDB 5VOT). We observed that the interaction spaces of Frss1l and Cacng2, which comprise the center of mass of all positions satisfying the cross-link distance restraint, partially overlap at the region close to the ligand-binding domain (LBD) of the AMPAR. In contrast, the interaction space of Olfm1 occupies the area close to the N-terminal domain (NTD) of the AMPAR. Similarly, we also found that the Frssl1 DOMON domain structure and the Cacng2 structure partially overlap in the docking models. Together, these results show that it is unlikely that Frss1l and Cacng2/8 interact simultaneously with the same AMPAR subunit, especially considering that the transmembrane domain of Frrs1l is not included in our interaction analysis (structure not determined). While this prediction is not experimentally confirmed, it is consistent with previous studies showing the spatial segregation of the Frrs1l- and Cacng2/8-containing AMPAR in the ER and synaptic membrane, respectively (*23*). We also provided an estimated binding location of AMPAR and Frrs1l, for which the binding site within the AMPAR interactor complex is currently unknown.

### Replication of cross-linking data and resource

A typical MS analysis with data-dependent acquisition usually achieves 70 to 80% reproducibility at the protein level in technical replicates and 60 to 70% at the peptide level (*24*), mainly due to the stochastic selection for sequencing of low-abundant proteins. To further discuss this issue and expand our cross-link repository, we performed cross-linking experiments of three additional biologically independent replicates of hippocampal synaptosomes. We obtained 7183 unique Lys-Lys cross-links comprising 1746 different proteins (dataset 2 in table S1A), of which 60% intraprotein cross-links and 63% cross-linked protein pairs (intra- and interprotein) were identified in at least two independent replicates (fig. S10A). We detected a low percentage of potentially false cross-links between cytoplasmic and extracellular regions (0.46%), similar to the first dataset (0.87%) (fig. S10B).

**Fig. 6:**
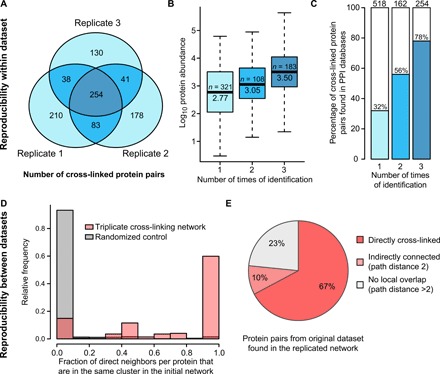
Evaluation of the XL-MS approach on three biologically independent replicates for hippocampal synaptosomes. (**A**) Venn diagram showing the number of identified cross-linked protein pairs (from interprotein cross-links) in different replicates (dataset 2). (**B**) Boxplot showing the distribution of protein abundances for cross-linked protein pairs identified in one, two, and all three replicates. The number of proteins and median abundance are indicated in each box. Protein abundance data were obtained from (*12*). (**C**) Percentage of cross-linked protein pairs present in any PPI databases (i.e., STRING, InWEB, and BioGRID) identified in one, two, and all three replicates. The total number of cross-linked protein pairs identified is indicated above each bar. (**D**) Distribution of the fraction of direct neighbors (cross-linked proteins pairs) from the triplicate dataset that are in the same cluster in the initial network (Fig. 3A, labeled in red) and a randomly rewired network (100 iterations, labeled in gray). (**E**) Pie chart showing the percentage of protein pairs from the initial hippocampal synaptosome dataset identified in the triplicate network. Only proteins containing interprotein cross-links in both datasets were considered.

To gain insight into the reproducibility of the XL-MS method within the same dataset, we inspected the overlap in interprotein cross-linked protein pairs of the individual replicates. Of the 934 protein pairs present in the dataset, 516 were found in only one, 162 in two, and 252 in three replicates (Fig. 6A). This is expected given the stochastic nature of the cross-linking events and low abundance of cross-linked peptide pairs. In accordance with our previous observations, the number of times a protein interaction was identified correlated to their abundances, with proteins present in all experimental replicates having more than five times higher median abundance than those found in only one sample (Fig. 6B). Furthermore, we examined the existence of the cross-links in public PPI databases. Of the 516 protein pairs that were identified in only one replicate, 32% corresponded to previously reported PPIs, while 78% of the 252 proteins pairs identified in all three replicates were reported PPIs (Fig. 6C). To assess reproducibility of the network topology between datasets, we compared the triplicate XL-MS dataset with the initial network (Fig. 3A). We observed that a large fraction of protein pairs in the replicated dataset belongs to the same clusters in the initial protein interaction network, showing a high similarity between the two datasets (two-sample Kolmogorov-Smirnov test, *P* = 2.2 × 10^−16^; Fig. 6D). Next, we compared the protein pairs from the initial hippocampal synaptosome preparation with the triplicate dataset considering only proteins present in both networks. We found 77% of the cross-linked protein pairs associated in the triplicate dataset, 67% directly and 10% indirectly (i.e., connected by one neighbor in common), showing a high reproducibility for proteins detected in both datasets (Fig. 6E). Together, these observations illustrate the reproducibility of XL-MS within and between datasets and indicate that cross-link identifications largely depend on the protein abundance, with proteins found in all experimental replicates being of highest abundance and mostly participating in previously reported PPIs.

Together, from the seven XL-MS experiments performed in this study, we obtained 11,999 unique cross-links comprising connections within and between 2362 proteins, representing one of the largest cross-linking datasets. All the cross-links identified are available for inspection in our interactive web tool (http://xlink.cncr.nl). The interaction network from all seven XL-MS experiments is also presented in fig. S11, including detailed information of each cross-link (e.g., the number of samples in which each PPI was found, identification FDR, and previously reported protein interactions). These data resource and online tools can assist the exploration and selection of cross-links of interest for further biochemical and/or biological follow-up experiments.

## DISCUSSION

In this study, we generated an extensive XL-MS dataset of synaptosomes and microsomes from mouse brains, yielding one of the largest cross-link collections to date (11,999 unique Lys-Lys connections). The complete dataset is available as a user-friendly resource for interrogation (http://xlink.cncr.nl). We used this recent type of data to investigate three different protein features, such as protein structure, protein interaction, and interaction sites, opening new avenues in revealing the architecture and assembly of protein complexes. For each of these features, we validated the reliability of the data by several approaches in key synaptic proteins, such as the structural dynamics of Camk2 and protein interaction sites of SNARE proteins. Last, we combined structural modeling and protein interaction site investigation based on XL-MS to extend our knowledge on the architecture of the AMPAR complex.

High-resolution protein structures provide a means to validate cross-link assignment and enable probing alternative conformational states of proteins/complexes. In line with our previous studies (*9*, *10*), we observed that the agreement between observed Lys-Lys distances and those predicted by high-resolution structures follows a highly distinctive bimodal distribution: Some proteins/complexes show at least 98% of matched cross-links, whereas others give a much lower value. In the presented data, >99% of the Lys-Lys distances in eight of nine protein complexes complied with the respective high-resolution structures, with the exception of Camk2, in which only about 65% of the cross-links were within the 23.4 + 10 Å distance restraint (in extended state). Whereas the organization of the rigid central hub of the holoenzyme has been elucidated, the relative positioning of the kinase domains remains challenging due to their high flexibility, especially in the extended and activation-competent conformation (*25*, *26*). Examination of the Camk2 structures of the extended, compact, and autoinhibited conformational states confirmed the existence of large movements (*26*) between the kinase and the hub domains via the extremely flexible linker region. Furthermore, alternative contacts between adjacent kinase domains were proposed on the basis of 11 long-distance cross-links centered at K258, a cross-linking hotspot at the kinase domain (Fig. 2C). In addition to other already reported interkinase domain interactions (*27*), these potential contacts show the dynamics of the kinase domains and extend our knowledge about their organization in-solution. Together, the exploration of three-dimensional protein structures allowed us to validate the reliability of our cross-linking approach and to probe the dynamic structure and the contacting possibilities for the kinase domains of Camk2.

In terms of PPIs, we generated an XL-based protein interaction network that revealed a highly modular organization and a scale-free topology, which has been shown to characterize many biological phenomena (*14*). Comparing our cross-linking data with the accumulated experimental evidences in STRING, InWEB and BioGRID databases showed that 39% of detected cross-links were previously reported high-confidence interactions, suggesting the presence of a large number novel candidate protein interactions in our dataset. Most proteins in each module participated in a shared cellular component and related protein complexes, such as the postsynaptic density, synaptic vesicles, G protein signaling, voltage-gated calcium channels, or the mitochondrial respiratory chain. The reverse was also found, i.e., proteins from a common cellular compartment or macromolecular machinery were found significantly more interconnected in our network. Similar results were observed for each brain area and subcellular fraction individually. The presence of diverse functional groups is in line with current models of the synapse arguing for high molecular and functional complexity (*6*), including protein synthesis, proteasomal degradation, mitochondrial function, and metabolic cascades. With further exploration, our cross-linking data may also provide additional insights into protein complexes involved in these biological processes.

Cross-linking technology has been previously used to assist the characterization of protein interaction surfaces (*8*, *28*), as was also suggested by the significant enrichment of interprotein cross-links within predicted interaction interfaces. To further validate interaction interfaces provided by our XL-MS, we used peptide array assays to resolve the binding sites for a selection of proteins of interest centered at the SNARE complex, as has been extensively used previously (*29*). The SNARE complex plays an essential role in presynaptic vesicle fusion leading to neurotransmitter release [reviewed in (*30*)]. We detected cross-links predominantly located in the Habc domain of Stx1a/b connected to other members of this molecular machinery, such as Stx1a, Stx1b, Stxbp1, and Snap25, well in agreement with the known protein complex topology (*31*). Whereas the majority of the XL-MS data are in good agreement with the binding sites determined by peptide array, we also found several cross-linking sites located at the proximity of the interaction interfaces but do not directly contribute to the binding, such as the two lysine residues of Stx1 (Lys45 and Lys55) detected between the Stxbp1-Stx1 interaction (*32*). These results confirmed that XL-MS provides a valuable exploratory platform for binding site prediction; however, this type of data ideally needs to be validated by orthogonal approaches (*28*).

Last, we used our XL-MS data to improve our understanding of protein interaction and assembly of the AMPAR complex. By modeling the interaction of Olfm1, Cacng2, Cacng8, and Frss1l with AMPAR, we found that the interaction space occupied by Olfm1 is mostly located at the NTD of AMPAR, whereas the interaction space of Cacng2 and the DOMON domain of Frss1l partly overlap at the AMPAR LBD. These analyses are in agreement with previous data obtained for the interaction of Olfm1 in the extracellular space (*33*) and the described binding sites of Cacng2/8 (*34*). In particular, the shared interaction space between Cacng2/8 and Frrs1l on the AMPAR suggests that their interactions with the AMPAR are exclusive and may occur as part of different protein complexes, in agreement with the mutually exclusive populations of AMPAR assemblies suggested by Brechet *et al*. (*23*). To our knowledge, these results present the first model on Frrs1l-AMPAR interaction, of which the loss has been shown to be critical for the development of epileptic-dyskinetic encephalopathy (*23*, *35*).

Several aspects of this recent XL-MS methodology were evaluated in this study. Regarding reliability, the statistical assessment of our data analysis software (*9*) estimated a 2% FDR. This value was supported by the topological validation, based on the cross-links found between cytoplasmic and extracellular regions, as well as the structural validation by measuring the distances of linked lysine pairs based on existing high-resolution structures. Regarding detection bias and coverage, we found that the main factor driving the detection of cross-links seems to be protein abundance. High-abundance proteins contained a higher number of cross-links and were present in a larger number of samples and replicates. This is likely due to the bias in MS2 selection during intensity-triggered data-dependent acquisitions (DDA), i.e., low abundance cross-links are less likely to be selected for sequencing in the mass spectrometer. No major bias was observed for the number of lysines of the protein or the subcellular distribution of the cross-linker, as a wide spectrum of synaptic proteins were cross-linked across the pre- and postsynaptic compartments, both in the cytoplasmic and extracellular regions. Regarding reproducibility, 60% of the intraprotein cross-links and 63% cross-linked protein pairs (intra- and interprotein) were identified in at least two independent replicates. These values are slightly lower than the typical reproducibility of 70 to 80% at the protein level and similar to the 60 to 70% reproducibility at the peptide level in LC-MS/MS experiments (*24*), consistently with the low abundance of cross-linked peptides. The similarity in protein-protein connectivity between datasets also argues in favor for the validity of the results obtained by XL-MS. In addition, a high reproducibility was observed for the proteins identified in both datasets, which indicates that cross-links not replicated are predominantly due to missing detections rather than to a different connectivity. Together, each cross-linking experiment provided data that corresponded to a partially stochastic subsample of the original protein interactions.

XL-MS provides valuable and complementary information in comparison to other techniques in protein interaction studies, such as proximity labeling and antibody-based proteomic approaches. The recently developed proximity labeling approaches rely on the identification of labeled proteins located in the vicinity of the protein of interest, which is engineered to contain an enzyme responsible for the labeling reaction (*36*). In these approaches, the labeling radius is based on the diffusion of the reactive molecule, which is difficult to control. Multiple filtering steps are required to improve the spatial resolution, which generally lead to the removal of more than 90% of the identified proteins. In contrast, XL-MS is a high-throughput methodology that defines the spatial resolution based on the length of the spacer arm of the cross-linker, thus providing a higher fidelity even in large-scale analysis. Antibody-based proteomics is a low-throughput approach usually focused on a few individual proteins and often presents a high background where typically 90% of proteins identified are unspecific interactors. Moreover, the success of the experiments critically depends on the availability of high-affinity and high-specificity antibodies, the strength of the protein interactions, and the efficiency of solubilization of the proteins. These limitations can be bypassed with XL-MS, which is capable of capturing weak and/or transient protein contacts from intact cells or organelles, as exemplified by the contacts between Camk2 kinase domains. Proximity labeling and antibody-based proteomic approaches only yield a list of protein identifications, while XL-MS provides maximum distance limits between residues. These residue-to-residue connections enable to distinguish direct and indirect protein interactions and provide a more detailed picture of the structural organization of protein complexes, as exemplified by the interaction space models of the AMPAR interactions. Conversely, XL-MS has a lower sensitivity compared with the other approaches, since cross-linked peptides are much less abundant than linear peptides, which may underlie the previous identification of a higher number of AMPAR interactors than the present study (*20*, *37*, *38*). In addition, XL-MS relies on the presence of amino acids susceptible to cross-link, which may allow interactions to be undetected. Future advances in mass spectrometer, in conjunction with the affinity isolation of the cross-linked peptides, hold the promise for increasing the sensitivity of the XL-MS approach.

Together, we applied XL-MS directly at subcellular fractions from mouse brain to open up new avenues in probing protein structures, assemblies, and interactions close to their native subcellular context. We extended the current knowledge on essential elements of the synapse, including Camk2 and the AMPAR, by a combination of structural modeling and protein interaction investigation. The reliability of the data was validated by several approaches, as we deemed necessary for this recent methodology. Given the molecular complexity of the synapse and the large amount and depth of the cross-linking data generated, additional hypotheses should emerge for other proteins and functional groups. To enable this exploration, we provided the complete dataset in table format (table S1) and as a user-friendly web-based platform (http://xlink.cncr.nl).

## MATERIALS AND METHODS

### Study design

The aim of our study was to reveal the architecture and assembly of synaptic protein complexes. We used XL-MS as basis to investigate protein structure, protein-protein interaction, and binding surfaces in their physiological subcellular context. We selected two subcellular fractions (synaptosomes and microsomes) from two different mouse brain regions (hippocampus and cerebellum) to extensively cover the synaptic proteome. Because of the discovery purpose of these experiments, each of the four preparations for MS analysis was performed as a single dataset (dataset 1). In addition, we performed cross-linking experiments of three biologically independent replicates of hippocampal synaptosomes to gain insight into the reproducibility of XL-MS and provide further data to be used as a resource (dataset 2). The fidelity of the results was extensively investigated by comparing the XL-MS data with previously reported high-resolution structures, PPIs, and binding sites. For peptide array interaction assays, two independent and two technical replicates were performed.

### Sample preparation

Ten mouse hippocampi and cerebellum were dissected from 8- to 10-week-old C57BL6 mice and stored at −80°C. The subcellular fractions were prepared as described previously (*12*). In brief, the brain regions were pooled and homogenized in a dounce homogenizer on ice [12 strokes, 900 revolutions per minute (rpm)] in homogenization buffer [0.32 M sucrose, 5 mM Hepes (pH 7.4), and protease inhibitor cocktail (Roche)]. The homogenate was centrifuged at 1000*g* for 10 min at 4°C, and the supernatant (S1) was divided for the different preparations. For the synaptosomal preparation, the supernatant (S1) was centrifuged at 100,000*g* for 2 hours at 4°C in a 0.85/1.2 M sucrose gradient. The synaptosomes were collected from the interface, diluted, and centrifuged at 18,000*g* for 20 min at 4°C. The pellet containing enriched synaptosomes was resuspended in homogenization buffer and kept for cross-linking. For the microsomal fraction, the supernatant (S1) was centrifuged at 20,000*g* for 20 min at 4°C, and the supernatant (S2) was ultracentrifuged at 100,000*g* for 2 hours at 4°C. The pellet containing enriched microsomes was resuspended in homogenization buffer and kept for the cross-linking procedure. All experiments were approved by the Vrije Universiteit Amsterdam Animal Users Care Committee and were performed in accordance with the relevant guidelines and regulations.

### Cross-linking and strong cation exchange fractionation

Cross-linking was performed by adding 1 mM DSSO (Thermo Fisher Scientific; resuspended freshly in anhydrous DMSO to 50 mM) and incubated at room temperature for 1 hour. The reaction was quenched by adding 50 mM tris buffer (pH 8.0) at room temperature for 30 min. The cross-linked samples were denatured with lysis buffer (8 M urea in 50 mM ammonium bicarbonate), reduced with 5 mM dithiothreitol at 56°C for 30 min, and alkylated with 15 mM iodoacetamide for 30 min in the dark at room temperature. Cross-linked proteins were digested with Lys-C for 4 hours at 37°C and subsequently digested by trypsin overnight. The resulting peptide mixture was desalted using Sep-Pak C18 cartridges (Waters), dried under vacuum, and fractionated by strong cation exchange (SCX) chromatography as previously described (*7*) to enrich higher-charged cross-linked peptides.

### LC-MS analysis

For each preparation, 20 to 40 SCX fractions containing predominantly higher-charged peptides (*z* ≥ 3) were analyzed by LC-MS using an ultrahigh-performance LC Agilent 1200 system (Agilent Technologies), equipped with an in-house packed C18 column for reversed-phase separation [column material: Poroshell 120 EC-C18, 2.7 μm (Agilent Technologies)] and coupled online to an Orbitrap Fusion mass spectrometer (Thermo Fisher Scientific). Mass analysis was performed using a previously described CID-MS2-MS3-ETD-MS2 acquisition strategy (*10*). Both MS1 and MS2 spectra were acquired in the Orbitrap mass analyzer, and MS3 spectra were acquired in the ion trap mass analyzer. Notably, MS3 acquisitions were only triggered when peak doublets with a specific mass difference (Δ = 31.9721 Da) were detected in the CID-MS2 spectra. The following MS parameters were used: MS1 resolution, 60,000; MS2 resolution, 30,000; MS2 isolation window, 1.6 *m*/*z*; MS3 isolation window, 3 *m*/*z*; MS2-CID normalized collision energy, 25%; and MS3-CID normalized collision energy, 35%; calibrated charge-dependent electron transfer dissociation (ETD) parameters were enabled.

### XL-MS data analysis

Peak lists (.mgf files) were generated in Proteome Discoverer (version 2.1) to convert each RAW file into three MGF files containing CID-MS2, ETD-MS2, and CID-MS3 data. During MGF conversion, the CID- and ETD-MS2 spectra were deconvoluted to charge state 1 using the MS2 Spectrum Processor add-on module in Proteome discoverer v2.1. The MGF files were used as input to identify cross-linked peptides with stand-alone XlinkX v2.0 (*10*). The following settings of XlinkX were used: MS ion mass tolerance, 10 parts per million (ppm); MS2 ion mass tolerance, 20 ppm; MS3 ion mass tolerance, 0.6 Da; fixed modification, Cys carbamidomethylation; variable modification, Met oxidation; enzymatic digestion, trypsin; and allowed number of missed cleavages, 3. All MS2 and MS3 spectra were searched against concatenated target-decoy databases generated based on the synapse proteome determined by bottom-up proteomics, containing 5133 target sequence entries. Cross-links were reported at 2% FDR based on a target-decoy calculation strategy (*10*). Cross-linked proteins identified in dataset 1 were annotated and evaluated using SynGO, with “brain expressed” gene set as background and 1% FDR (table S1B). Raw data were deposited in the PRIDE repository with the identifier PXD010317 and PXD015160. All identified cross-links can be accessed in table S1 and via our web-based tool (http://xlink.cncr.nl) for further investigations.

### Structural modeling

The following structures were used for the spatial distance measurements: the AMPA-type glutamate receptor (homomeric Gria2; only cross-links unique to Gria2 were used; PDB 3KG2), the voltage-dependent calcium channel (PDB 5GJV), the vesicular adenosine triphosphatase (PDB 3J9V), the mitochondrial electron transport chain complexes I to IV (PDB 5LNK, 1ZOY, 1NTM, and 1 V54), and the 80*S* ribosome (PDB 4UG0). The Gria2 homomeric structure was used since this subunit contained the highest number of cross-links compared with the other Gria proteins (i.e., Gria1, Gria3, and Gria4).

The different conformational states of Camk2 were modeled on the basis of the structures of the extended Camk2 structure of rat (PDB 5U6Y), the autoinhibited kinase domain of *Caenorhabditis elegans* (PDB 2BDW), and the compact conformation of human (PDB 3SOA). Homology modeling was performed with Maestro 11.4 (Schrödinger, LLC, New York, NY, USA). All manual docking, manipulation, optimization, and loop generation (G315-V345 for autoinhibited and T305-V345 for compact conformation) were created using the Loop Search tool implemented in SYBYL-X 2.1.1 (Certara USA Inc., St. Louis, MO, USA). Energy minimization was performed using AMBER7 FF99 force field. Structure images and morphs between the different conformations were generated with PyMOL 1.8.4.1.

The following available structures were used in the structural analysis of the AMPAR auxiliary protein complex: PDB 5IDE for AMPAR, PDB 5VOT for Cacng2/8, and PDB 5AMO for Olfm1. The structure of the DOMON domain of Frss1l was modeled on the basis of the sequence homology using the available structure with the highest sequence coverage (PDB 4ZEL). The side chains of AMPAR were modeled with Scwrl4, and the missing loop of Cacng2 (residues 39 to 56) was modeled with I-TASSER. Interaction space models were calculated using the DisVis Webserver, using a maximal Cα-Cα distance of 23.4 Å. Docking models were generated using HADDOCK2.2 for the Frrs1l DOMON domain and Olfm1. Cacng2 was structurally aligned in PyMOL from PDB 5VOT.

### Protein network analysis

The XL-based PPI network was generated on the basis of interprotein cross-links and visualized using Cytoscape v3.4.0 for each subcellular fraction and brain area, as well as for the different conditions combined. Myelin basic protein, which presented a high number of cross-links, was removed from the network to improve the readability of the figures, and its cross-links can be found in the table S1, our web-based platform, and fig. S11. Unsupervised edge-betweenness clustering was applied to the core component of the network, and each cluster was annotated according to the Cellular Component of the GO enrichment analysis, with all proteins identified as background (complete analysis and exceptions shown are in table S2B). Disconnected modules were grouped using DAVID Gene Functional Classification using medium and low stringency, and each group was subsequently annotated. The connectivity between annotated proteins was tested by measuring the network path distances and the number of protein pairs directly connected with the same GO annotation (for cellular component terms with >50 proteins in the core component of the network). For the latter measurement, we performed graph rewiring while preserving the degree distribution (repeated in 100 permutations) as a control using the igraph R package. For each individual network and the replication experiment, this analysis was performed using the clusters from the combined network. The same package was used to calculate the modularity score of the network. Protein abundances were obtained from our previous proteomic study of synaptosomes and microsomes (*12*). Protein domain information of all cross-linked proteins in our dataset was retrieved from UniProt (table S1, A and C). Protein interactions known from literature were obtained from three databases: STRING (*15*), BioGRID (*17*), and InWEB (*16*). Only experimentally determined interactions and physical interactions were considered from STRING and BioGRID, respectively. For classification of confidence levels, a combined score of <400 was considered as low, 400 to 700 as medium, and ≥700 as high for STRING; a final score of <0.2 or no score provided was considered as low, 0.2 to 0.7 as medium, and ≥0.7 as high for InWEB; interactions evidenced by low-throughput experiments were considered as high and the rest as low confidence for BioGRID. Only interactions with high-confidence level in at least one database were considered for downstream analysis (i.e., comparisons shown in Fig. 3, C and D).

### Expression plasmids

Mouse *Stx1a* (NM_016801) was cloned into the pCMV6-Entry vector, yielding Stx1a-pCMV6-Entry including a C-terminal Myc-Flag tag (OriGene MR203927). *Snap25* (NM_030991.3) was Gateway cloned into pDEST–EGFP (enhanced green fluorescent protein) vector, yielding Snap25-pDEST-EGFP including three N-terminal Flag tags. *Stxbp1* (NM_013038.3) was Gateway cloned into pDEST vector, yielding Stxbp1-pDEST including three N-terminal Flag tags. Mouse *Stxbp5* and *Stxbp5l* were fused to EYFP (enhanced yellow fluorescent protein) tag and cloned into a p156RRL vector, as described previously (*39*).

### Cell culture and transfection

HEK293 cells (the American Type Culture Collection, ATCC) were cultured in 10-cm dishes as described previously (*40*). Cells were transfected at 60% confluency with 5 μg of plasmid using polyethylenimine (25 kDa linear, Polysciences) and incubated for 48 hours. Cells were washed twice with ice-cold phosphate-buffered saline and collected in extraction buffer [0.5% n-Dodecyl b-d-maltoside (DDM), 25 mM Hepes (pH 7.4), 150 mM NaCl, and EDTA-free complete protease inhibitor (Roche)]. Proteins were extracted by gently mixing at 10 rpm for 1 hour at 4°C and separated from the insoluble debris by two centrifugation steps for 20 min at 20,000*g*.

### Protein interaction interfaces analysis

The position of the mouse cross-linked lysines was mapped onto the human proteome as retrieved from UniProt. Only lysines with high-confidence mapping were considered for further analysis, i.e., lysine located at the same protein position with identical amino acid sequence up to the lysine position. The corresponding cross-linked lysines in human were compared to human protein interaction interfaces based on Interactome INSIDER (*18*). Enrichment analysis was performed by Fisher’s exact test.

### Peptide array interaction assays

CelluSpots peptide arrays (Intavis Bioanalytical Instruments AG) were blocked with 3% bovine serum albumin (Sigma-Aldrich) in TBS-T (25 mM tris, 150 mM NaCl, and 0.005% Tween 20, pH 7.4) for 1 hour. After three washes with TBS-T, the arrays were incubated with protein extracts from expressing cells overnight at 4°C (one 10-cm culture dish for each array). Next, arrays were washed three times and incubated with primary antibodies (Flag or GFP tag for different proteins) for 2 hours at 4°C. IRDye 800 secondary antibody was used for the detection using an Odyssey Fc imaging system (LI-COR Biosciences). Images were obtained with Image Studio software (version 2.0.38) and analyzed with Protein Array Analyzer for ImageJ. The background was subtracted, and signals were normalized to the positive control spot of each array (antibody antigen). The arithmetic mean of the three spots with protein tags not present in the proteins probed (Myc, His, and hemagglutinin HA) was used as negative control. The two technical replicates performed for each protein were averaged, followed by the subtraction of the signals of nontransfected (for Flag-tagged proteins) or GFP (for GFP-fused proteins) nonspecific binding controls. For low-signal spots, only the overlapping protein sequences of at least two different peptides with signals after the filtering were considered as positive hits. The following antibodies were used for the assay: Flag-tag (1:1000, Sigma-Aldrich, F1804), GFP (1:2000, NeuroMab, 75-131), and IRDye 800CW goat anti-mouse (1:10,000, LI-COR Biotechnology, 925-32210).

## Supplementary Material

http://advances.sciencemag.org/cgi/content/full/6/8/eaax5783/DC1

Download PDF

Table S1A

Table S2

Table S3

Table S4

Movie S1

Stitching the synapse: Cros-linking mas spectrometry into resolving synaptic protein interactions

## SUPPLEMENTARY MATERIALS

Supplementary material for this article is available at http://advances.sciencemag.org/cgi/content/full/6/8/eaax5783/DC1 Fig. S1. General evaluation of the XL-MS approach. Fig. S2. Mapping of cross-linking data onto high-resolution structure of several protein complexes. Fig. S3. Characterization of XL-based protein interaction network from hippocampus synaptosomes. Fig. S4. Characterization of XL-based protein interaction network from hippocampus microsomes. Fig. S5. Characterization of XL-based protein interaction network from cerebellum synaptosomes. Fig. S6. Characterization of XL-based protein interaction network from cerebellum microsomes. Fig. S7. Extended and detailed XL-based protein interaction network (extended Fig. 3A). Fig. S8. XL-based protein interaction network analysis. Fig. S9. Protein interaction interfaces and peptide array analysis (extended Fig. 4). Fig. S10. Evaluation of XL-MS approach on biologically independent replicates for hippocampal synaptosome (extended Fig. 6). Fig. S11. Complete XL-based protein interaction network from all seven cross-linking MS experiments. Table S1A. Complete list of cross-links identified in the two datasets. Table S1B. SynGO enrichment analysis of proteins identified in dataset 1. Table S1C. Cross-linked protein list. Table S2. Clustering and GO enrichment analysis of the proteins in the XL-based protein interaction network. Table S3. Human protein mapping and overlap of cross-linked lysine positions with protein interaction interfaces. Table S4. Sequences and signal intensities for the peptides included in the two independent replicates of peptide arrays (fig. S9B). Movie S1. Dynamic simulation of the three conformational states of Camk2.

## Appendix

View/request a protocol for this paper from *Bio-protocol*.

## References

[1] Mayford M., Siegelbaum S. A., Kandel E. R., Synapses and memory storage. Cold Spring Harb. Perspect. Biol. 4, a005751 (2012). 22496389 10.1101/cshperspect.a005751 PMC3367555

[2] Grant S. G. N., Synaptopathies: Diseases of the synaptome. Curr. Opin. Neurobiol. 22, 522–529 (2012). 22409856 10.1016/j.conb.2012.02.002

[3] Bayés A., Grant S. G. N., Neuroproteomics: Understanding the molecular organization and complexity of the brain. Nat. Rev. Neurosci. 10, 635–646 (2009). 19693028 10.1038/nrn2701

[4] Chua J. J. E., Kindler S., Boyken J., Jahn R., The architecture of an excitatory synapse. J. Cell Sci. 123, 819–823 (2010). 20200227 10.1242/jcs.052696

[5] Li K. W., Klemmer P., Smit A. B., Interaction proteomics of synapse protein complexes. Anal. Bioanal. Chem. 397, 3195–3202 (2010). 20361179 10.1007/s00216-010-3658-z PMC2911543

[6] Dieterich D. C., Kreutz M. R., Proteomics of the synapse – a quantitative approach to neuronal plasticity. Mol. Cell. Proteomics 15, 368–381 (2016). 26307175 10.1074/mcp.R115.051482 PMC4739661

[7] Liu F., Rijkers D. T. S., Post H., Heck A. J. R., Proteome-wide profiling of protein assemblies by cross-linking mass spectrometry. Nat. Methods 12, 1179–1184 (2015). 26414014 10.1038/nmeth.3603

[8] Liu F., Heck A. J. R., Interrogating the architecture of protein assemblies and protein interaction networks by cross-linking mass spectrometry. Curr. Opin. Struct. Biol. 35, 100–108 (2015). 26615471 10.1016/j.sbi.2015.10.006

[9] Liu F., Lössl P., Rabbitts B. M., Balaban R. S., Heck A. J. R., The interactome of intact mitochondria by cross-linking mass spectrometry provides evidence for coexisting respiratory supercomplexes. Mol. Cell. Proteomics 17, 216–232 (2018). 29222160 10.1074/mcp.RA117.000470 PMC5795388

[10] Liu F., Lössl P., Scheltema R., Viner R., Heck A. J. R., Optimized fragmentation schemes and data analysis strategies for proteome-wide cross-link identification. Nat. Commun. 8, 15473 (2017). 28524877 10.1038/ncomms15473 PMC5454533

[11] Koopmans F., van Nierop P., Andres-Alonso M., Byrnes A., Cijsouw T., Coba M. P., Cornelisse L. N., Farrell R. J., Goldschmidt H. L., Howrigan D. P., Hussain N. K., Imig C., de Jong A. P. H., Jung H., Kohansalnodehi M., Kramarz B., Lipstein N., Lovering R. C., MacGillavry H., Mariano V., Mi H., Ninov M., Osumi-Sutherland D., Pielot R., Smalla K. H., Tang H., Tashman K., Toonen R. F. G., Verpelli C., Reig-Viader R., Watanabe K., van Weering J., Achsel T., Ashrafi G., Asi N., Brown T. C., De Camilli P., Feuermann M., Foulger R. E., Gaudet P., Joglekar A., Kanellopoulos A., Malenka R., Nicoll R. A., Pulido C., de Juan-Sanz J., Sheng M., Südhof T. C., Tilgner H. U., Bagni C., Bayés À., Biederer T., Brose N., Chua J. J. E., Dieterich D. C., Gundelfinger E. D., Hoogenraad C., Huganir R. L., Jahn R., Kaeser P. S., Kim E., Kreutz M. R., McPherson P. S., Neale B. M., O’Connor V., Posthuma D., Ryan T. A., Sala C., Feng G., Hyman S. E., Thomas P. D., Smit A. B., Verhage M., SynGO: An evidence-based, expert-curated knowledge base for the synapse. Neuron 103, 217–234.e4 (2019). 31171447 10.1016/j.neuron.2019.05.002 PMC6764089

[12] Pandya N. J., Koopmans F., Slotman J. A., Paliukhovich I., Houtsmuller A. B., Smit A. B., Li K. W., Correlation profiling of brain sub-cellular proteomes reveals co-assembly of synaptic proteins and subcellular distribution. Sci. Rep. 7, 12107 (2017). 28935861 10.1038/s41598-017-11690-3 PMC5608747

[13] Merkley E. D., Rysavy S., Kahraman A., Hafen R. P., Daggett V., Adkins J. N., Distance restraints from crosslinking mass spectrometry: Mining a molecular dynamics simulation database to evaluate lysine-lysine distances. Protein Sci. 23, 747–759 (2014). 24639379 10.1002/pro.2458 PMC4093951

[14] Vidal M., Cusick M. E., Barabási A.-L., Interactome networks and human disease. Cell 144, 986–998 (2011). 21414488 10.1016/j.cell.2011.02.016 PMC3102045

[15] Szklarczyk D., Franceschini A., Wyder S., Forslund K., Heller D., Huerta-Cepas J., Simonovic M., Roth A., Santos A., Tsafou K. P., Kuhn M., Bork P., Jensen L. J., von Mering C., STRING v10: Protein–protein interaction networks, integrated over the tree of life. Nucleic Acids Res. 43, D447–D452 (2015). 25352553 10.1093/nar/gku1003 PMC4383874

[16] Li T., Wernersson R., Hansen R. B., Horn H., Mercer J., Slodkowicz G., Workman C. T., Rigina O., Rapacki K., Stærfeldt H. H., Brunak S., Jensen T. S., Lage K., A scored human protein-protein interaction network to catalyze genomic interpretation. Nat. Methods 14, 61–64 (2017). 27892958 10.1038/nmeth.4083 PMC5839635

[17] Stark C., Breitkreutz B. J., Reguly T., Boucher L., Breitkreutz A., Tyers M., BioGRID: A general repository for interaction datasets. Nucleic Acids Res. 34, D535–D539 (2006). 16381927 10.1093/nar/gkj109 PMC1347471

[18] Meyer M. J., Beltrán J. F., Liang S., Fragoza R., Rumack A., Liang J., Wei X., Yu H., Interactome INSIDER: A structural interactome browser for genomic studies. Nat. Methods 15, 107–114 (2018). 29355848 10.1038/nmeth.4540 PMC6026581

[19] Christie M. P., Whitten A. E., King G. J., Hu S. H., Jarrott R. J., Chen K. E., Duff A. P., Callow P., Collins B. M., James D. E., Martin J. L., Low-resolution solution structures of Munc18:Syntaxin protein complexes indicate an open binding mode driven by the Syntaxin N-peptide. Proc. Natl. Acad. Sci. U.S.A. 109, 9816–9821 (2012). 22670057 10.1073/pnas.1116975109 PMC3382502

[20] Schwenk J., Harmel N., Brechet A., Zolles G., Berkefeld H., Müller C. S., Bildl W., Baehrens D., Hüber B., Kulik A., Klöcker N., Schulte U., Fakler B., High-resolution proteomics unravel architecture and molecular diversity of native AMPA receptor complexes. Neuron 74, 621–633 (2012). 22632720 10.1016/j.neuron.2012.03.034

[21] Payne H. L., The role of transmembrane AMPA receptor regulatory proteins (TARPs) in neurotransmission and receptor trafficking (Review). Mol. Membr. Biol. 25, 353–362 (2008). 18446621 10.1080/09687680801986480

[22] Martin L. J., Blackstone C. D., Levey A. I., Huganir R. L., Price D. L., AMPA glutamate receptor subunits are differentially distributed in rat brain. Neuroscience 53, 327–358 (1993). 8388083 10.1016/0306-4522(93)90199-p

[23] Brechet A., Buchert R., Schwenk J., Boudkkazi S., Zolles G., Siquier-Pernet K., Schaber I., Bildl W., Saadi A., Bole-Feysot C., Nitschke P., Reis A., Sticht H., al-Sanna’a N., Rolfs A., Kulik A., Schulte U., Colleaux L., Abou Jamra R., Fakler B., AMPA-receptor specific biogenesis complexes control synaptic transmission and intellectual ability. Nat. Commun. 8, 15910 (2017). 28675162 10.1038/ncomms15910 PMC5500892

[24] Tabb D. L., Vega-Montoto L., Rudnick P. A., Variyath A. M., Ham A.-J. L., Bunk D. M., Kilpatrick L. E., Billheimer D. D., Blackman R. K., Cardasis H. L., Carr S. A., Clauser K. R., Jaffe J. D., Kowalski K. A., Neubert T. A., Regnier F. E., Schilling B., Tegeler T. J., Wang M., Wang P., Whiteaker J. R., Zimmerman L. J., Fisher S. J., Gibson B. W., Kinsinger C. R., Mesri M., Rodriguez H., Stein S. E., Tempst P., Paulovich A. G., Liebler D. C., Spiegelman C., Repeatability and reproducibility in proteomic identifications by liquid chromatography-tandem mass spectrometry. J. Proteome Res. 9, 761–776 (2010). 19921851 10.1021/pr9006365 PMC2818771

[25] Chao L. H., Stratton M. M., Lee I.-H., Rosenberg O. S., Levitz J., Mandell D. J., Kortemme T., Groves J. T., Schulman H., Kuriyan J., A mechanism for tunable autoinhibition in the structure of a human Ca^2+^/calmodulin- dependent kinase II holoenzyme. Cell 146, 732–745 (2011). 21884935 10.1016/j.cell.2011.07.038 PMC3184253

[26] Myers J. B., Zaegel V., Coultrap S. J., Miller A. P., Bayer K. U., Reichow S. L., The CaMKII holoenzyme structure in activation-competent conformations. Nat. Commun. 8, 15742 (2017). 28589927 10.1038/ncomms15742 PMC5467236

[27] Chao L. H., Pellicena P., Deindl S., Barclay L. A., Schulman H., Kuriyan J., Intersubunit capture of regulatory segments is a component of cooperative CaMKII activation. Nat. Struct. Mol. Biol. 17, 264–272 (2010). 20139983 10.1038/nsmb.1751 PMC2855215

[28] Liu Q., Remmelzwaal S., Heck A. J. R., Akhmanova A., Liu F., Facilitating identification of minimal protein binding domains by cross-linking mass spectrometry. Sci. Rep. 7, 13453 (2017). 29044157 10.1038/s41598-017-13663-y PMC5647383

[29] Katz C., Levy-Beladev L., Rotem-Bamberger S., Rito T., Rüdiger S. G. D., Friedler A., Studying protein–protein interactions using peptide arrays. Chem. Soc. Rev. 40, 2131–2145 (2011). 21243154 10.1039/c0cs00029a

[30] Südhof T. C., Rothman J. E., Membrane fusion: Grappling with SNARE and SM proteins. Science 323, 474–477 (2009). 19164740 10.1126/science.1161748 PMC3736821

[31] Misura K. M. S., Scheller R. H., Weis W. I., Three-dimensional structure of the neuronal-Sec1-syntaxin 1a complex. Nature 404, 355–362 (2000). 10746715 10.1038/35006120

[32] Burkhardt P., Hattendorf D. A., Weis W. I., Fasshauer D., Munc18a controls SNARE assembly through its interaction with the syntaxin N-peptide. EMBO J. 27, 923–933 (2008). 18337752 10.1038/emboj.2008.37 PMC2323264

[33] Pandya N. J., Seeger C., Babai N., Gonzalez-Lozano M. A., Mack V., Lodder J. C., Gouwenberg Y., Mansvelder H. D., Danielson U. H., Li K. W., Heine M., Spijker S., Frischknecht R., Smit A. B., Noelin1 affects lateral mobility of synaptic AMPA receptors. Cell Rep. 24, 1218–1230 (2018). 30067977 10.1016/j.celrep.2018.06.102 PMC6088136

[34] Cais O., Herguedas B., Krol K., Cull-Candy0 S. G., Farrant M., Greger I. H., Mapping the interaction sites between AMPA receptors and TARPs reveals a role for the receptor N-terminal domain in channel gating. Cell Rep. 9, 728–740 (2014). 25373908 10.1016/j.celrep.2014.09.029 PMC4405707

[35] Madeo M., Stewart M., Sun Y., Sahir N., Wiethoff S., Chandrasekar I., Yarrow A., Rosenfeld J. A., Yang Y., Cordeiro D., McCormick E. M., Muraresku C. C., Jepperson T. N., McBeth L. J., Seidahmed M. Z., el Khashab H. Y., Hamad M., Azzedine H., Clark K., Corrochano S., Wells S., Elting M. W., Weiss M. M., Burn S., Myers A., Landsverk M., Crotwell P. L., Waisfisz Q., Wolf N. I., Nolan P. M., Padilla-Lopez S., Houlden H., Lifton R., Mane S., Singh B. B., Falk M. J., Mercimek-Mahmutoglu S., Bilguvar K., Salih M. A., Acevedo-Arozena A., Kruer M. C., Loss-of-function mutations in FRRS1L lead to an epileptic-dyskinetic encephalopathy. Am. J. Hum. Genet. 98, 1249–1255 (2016). 27236917 10.1016/j.ajhg.2016.04.008 PMC4908178

[36] Han S., Li J., Ting A. Y., Proximity labeling: Spatially resolved proteomic mapping for neurobiology. Curr. Opin. Neurobiol. 50, 17–23 (2018). 29125959 10.1016/j.conb.2017.10.015 PMC6726430

[37] Schwenk J., Baehrens D., Haupt A., Bildl W., Boudkkazi S., Roeper J., Fakler B., Schulte U., Regional diversity and developmental dynamics of the AMPA-receptor proteome in the mammalian brain. Neuron 84, 41–54 (2014). 25242221 10.1016/j.neuron.2014.08.044

[38] Chen N., Pandya N. J., Koopmans F., Castelo-Székelv V., van der Schors R. C., Smit A. B., Li K. W., Interaction proteomics reveals brain region-specific AMPA receptor complexes. J. Proteome Res. 13, 5695–5706 (2014). 25337787 10.1021/pr500697b

[39] Geerts C. J., Mancini R., Chen N., Koopmans F. T. W., Li K. W., Smit A. B., van Weering J. R. T., Verhage M., Groffen A. J. A., Tomosyn associates with secretory vesicles in neurons through its N- and C-terminal domains. PLOS ONE 12, e0180912 (2017). 28746398 10.1371/journal.pone.0180912 PMC5529015

[40] Pandya N. J., Klaassen R. V., van der Schors R. C., Slotman J. A., Houtsmuller A., Smit A. B., Li K. W., Group 1 metabotropic glutamate receptors 1 and 5 form a protein complex in mouse hippocampus and cortex. Proteomics 16, 2698–2705 (2016). 27392515 10.1002/pmic.201500400 PMC5129514

