# Supplementary material for: Stitching the synapse: Cross-linking mass spectrometry into resolving synaptic protein interactions
Source: Sci Adv. 2020 Feb 19;6(8):eaax5783. doi: 10.1126/sciadv.aax5783 (PMC7030922; doi:10.1126/sciadv.aax5783)
Supplement: http://advances.sciencemag.org/cgi/content/full/6/8/eaax5783/DC1 [file supp_6_8_eaax5783__index.html]

Science Advances | Science AdvancesAAASSearchScience AdvancesMenu

## Supplementary Materials

**The PDF file includes:**

- Fig. S1. General evaluation of the XL-MS approach.
- Fig. S2. Mapping of cross-linking data onto high-resolution structure of several protein complexes.
- Fig. S3. Characterization of XL-based protein interaction network from hippocampus synaptosomes.
- Fig. S4. Characterization of XL-based protein interaction network from hippocampus microsomes.
- Fig. S5. Characterization of XL-based protein interaction network from cerebellum synaptosomes.
- Fig. S6. Characterization of XL-based protein interaction network from cerebellum microsomes.
- Fig. S7. Extended and detailed XL-based protein interaction network (extended Fig. 3A).
- Fig. S8. XL-based protein interaction network analysis.
- Fig. S9. Protein interaction interfaces and peptide array analysis (extended Fig. 4).
- Fig. S10. Evaluation of XL-MS approach on biologically independent replicates for hippocampal synaptosome (extended Fig. 6).
- Fig. S11. Complete XL-based protein interaction network from all seven cross-linking MS experiments.

Download PDF

**Other Supplementary Material for this manuscript includes the following:**

- Table S1A (Microsoft Excel format). Complete list of cross-links identified in the two datasets.
- Table S1B (Microsoft Excel format). SynGO enrichment analysis of proteins identified in dataset 1.
- Table S1C (Microsoft Excel format). Cross-linked protein list.
- Table S2 (Microsoft Excel format). Clustering and GO enrichment analysis of the proteins in the XL-based protein interaction network.
- Table S3 (Microsoft Excel format). Human protein mapping and overlap of cross-linked lysine positions with protein interaction interfaces.
- Table S4 (Microsoft Excel format). Sequences and signal intensities for the peptides included in the two independent replicates of peptide arrays (fig. S9B).
- Movie S1 (.mp4 format). Dynamic simulation of the three conformational states of Camk2.

**Files in this Data Supplement:**

- Adobe PDF - aax5783\_SM.pdf
