# Supplementary material for: Stitching the synapse: Cross-linking mass spectrometry into resolving synaptic protein interactions
Source: Sci Adv. 2020 Feb 19;6(8):eaax5783. doi: 10.1126/sciadv.aax5783 (PMC7030922; doi:10.1126/sciadv.aax5783)
Supplement: Download PDF [file aax5783_SM.pdf]

Science Advances
AAAS

advances.sciencemag.org/cgi/content/full/6/8/eaax5783/DC1

Supplementary Materials for

Stitching the synapse: Cross-linking mass spectrometry into resolving synaptic protein interactions

M. A. Gonzalez-Lozano, F. Koopmans, P. F. Sullivan, J. Protze, G. Krause, M. Verhage, K. W. Li*, F. Liu*, A. B. Smit*

*Corresponding author. Email: k.w.li@vu.nl (K.W.L.); fliu@fmp-berlin.de (F.L.); guus.smit@vu.nl (A.B.S.)

Published 19 February 2020, *Sci. Adv.* **6**, eaax5783 (2020)
DOI: 10.1126/sciadv.aax5783

The PDF file includes:

Fig. S1. General evaluation of the XL-MS approach.
Fig. S2. Mapping of cross-linking data onto high-resolution structure of several protein complexes.
Fig. S3. Characterization of XL-based protein interaction network from hippocampus synaptosomes.
Fig. S4. Characterization of XL-based protein interaction network from hippocampus microsomes.
Fig. S5. Characterization of XL-based protein interaction network from cerebellum synaptosomes.
Fig. S6. Characterization of XL-based protein interaction network from cerebellum microsomes.
Fig. S7. Extended and detailed XL-based protein interaction network (extended Fig. 3A).
Fig. S8. XL-based protein interaction network analysis.
Fig. S9. Protein interaction interfaces and peptide array analysis (extended Fig. 4).
Fig. S10. Evaluation of XL-MS approach on biologically independent replicates for hippocampal synaptosome (extended Fig. 6).
Fig. S11. Complete XL-based protein interaction network from all seven cross-linking MS experiments.

Other Supplementary Material for this manuscript includes the following:

(available at advances.sciencemag.org/cgi/content/full/6/8/eaax5783/DC1)

Table S1A (Microsoft Excel format). Complete list of cross-links identified in the two datasets.
Table S1B (Microsoft Excel format). SynGO enrichment analysis of proteins identified in dataset 1.
Table S1C (Microsoft Excel format). Cross-linked protein list.

Table S2 (Microsoft Excel format). Clustering and GO enrichment analysis of the proteins in the XL-based protein interaction network.
Table S3 (Microsoft Excel format). Human protein mapping and overlap of cross-linked lysine positions with protein interaction interfaces.
Table S4 (Microsoft Excel format). Sequences and signal intensities for the peptides included in the two independent replicates of peptide arrays (fig. S9B).
Movie S1 (.mp4 format). Dynamic simulation of the three conformational states of Camk2.

Supplementary figures

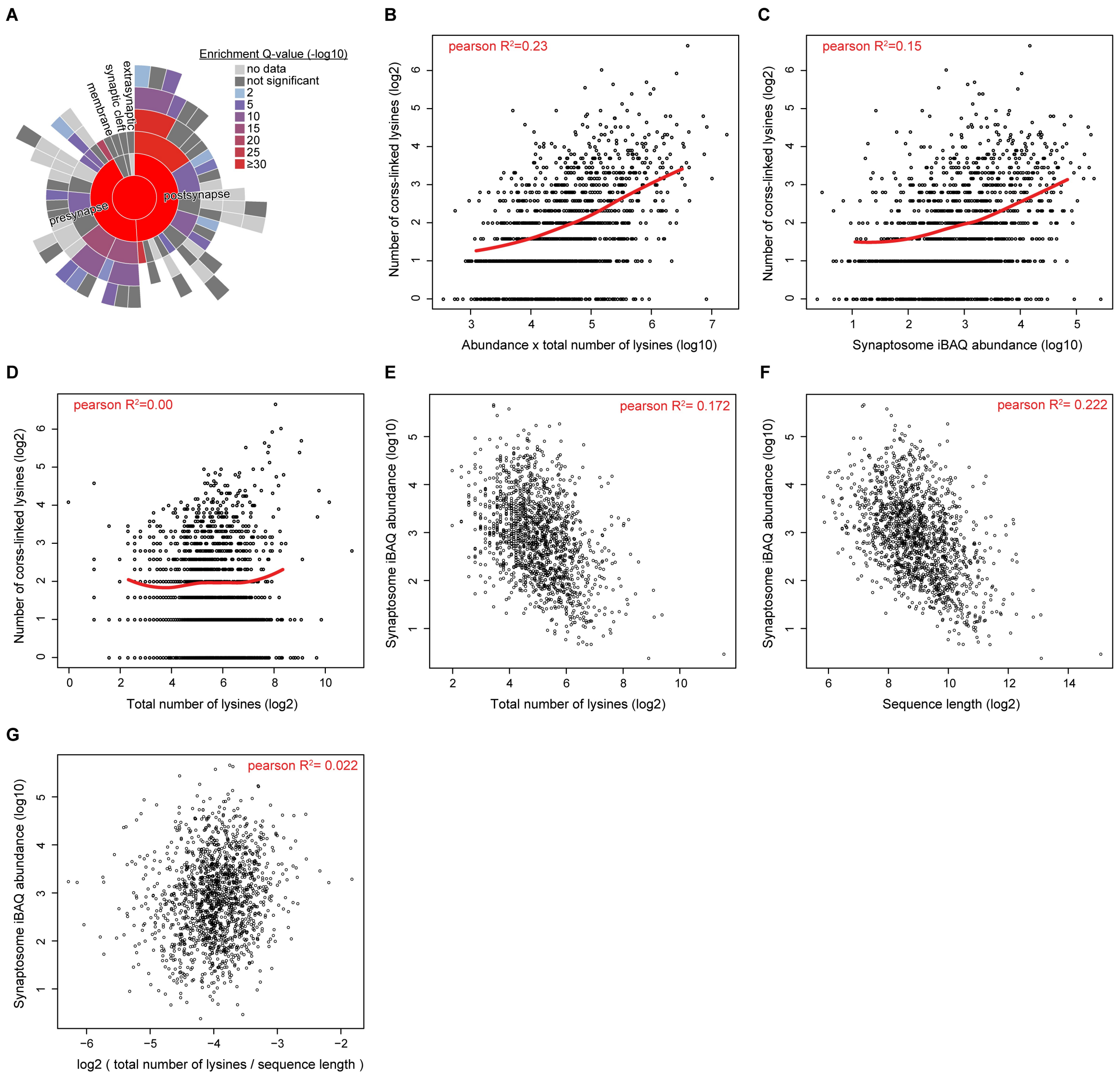

Fig. S1. General evaluation of the XL-MS approach. (**A**) Sunburst plot showing the annotation in synaptic functions of the cross-linked proteins identified (cellular component SynGO terms (*11*)). Inner rings are parent terms of more specific child terms in outer rings, color-coded according to enrichment Q-value. Notably, a wide and significant coverage of synapse-specific proteins is found distributed across both pre- and post-synapse locations. (**B**) Correlation between the number of cross-linked lysines of each protein and the protein abundance corrected for the total number of lysines, (**C**) the protein abundance, and (**D**) the total number of lysines. (**E-G**) Correlation between the protein abundance of each protein and the total number of lysines or sequence length.

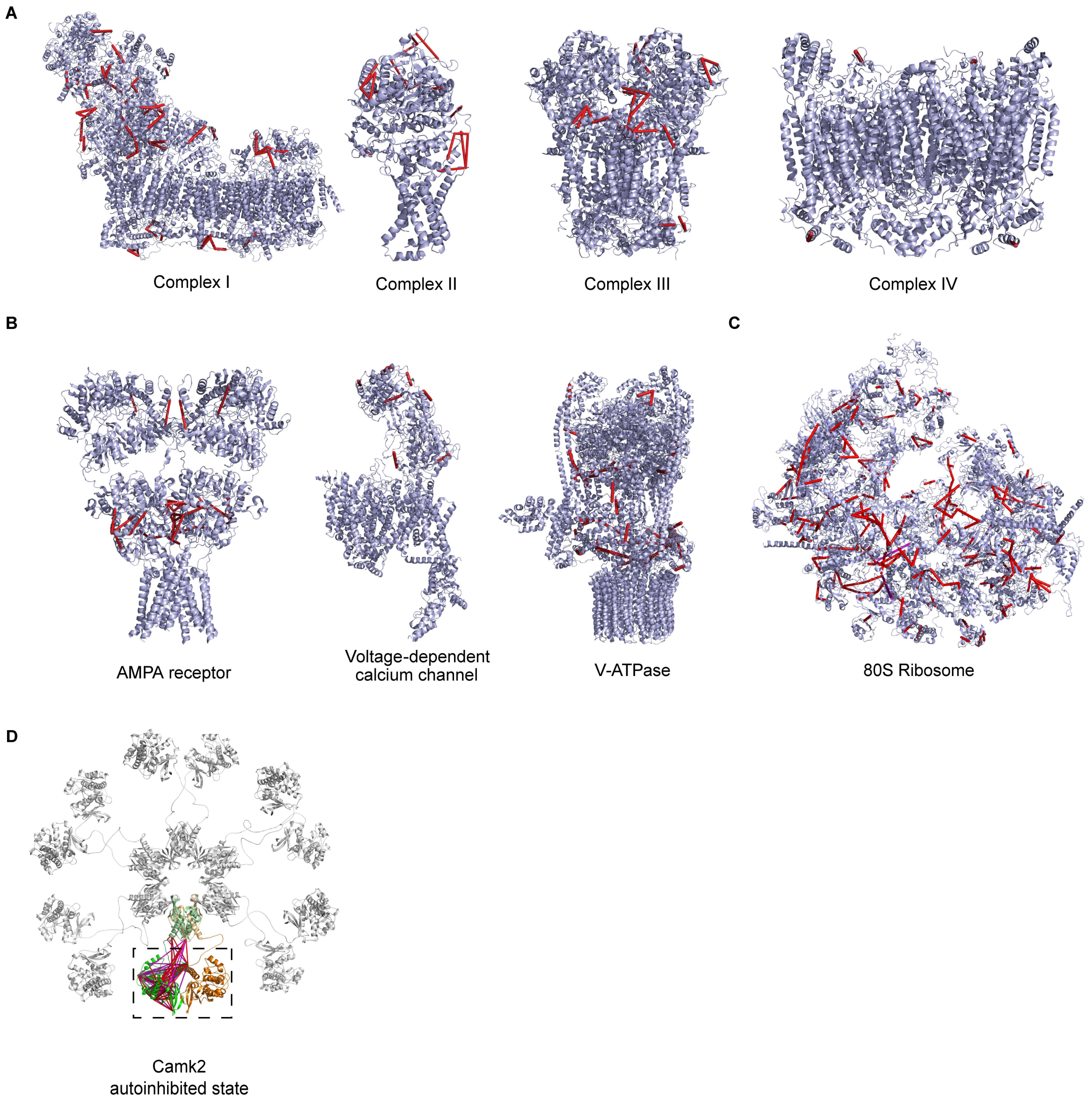

Fig. S2. Mapping of cross-linking data onto high-resolution structure of several protein complexes. (**A**) Cross-link mapping of the mitochondrial electron transport chain (ETC) complexes I-IV (PDB 5LNK, 1ZOY, 1NTM and 1V54). (**B**) Cross-link mapping of three selected synaptic protein complexes: the AMPA receptor (PDB 3KG2), voltage-dependent calcium channel (VDCC, PDB 5GJV), and vacuolar-type ATPase (V-ATPase, PDB 3J9V). (**C**) Cross-link mapping of the 80S ribosome (PDB 4UG0). For clarity, nucleic acids are not shown. (**D**) Full length model of an auto-inhibited state of Camk2 with high-resolution structure of the auto-inhibited kinase dimer (dotted rectangle, PDB 2BDW). Cross-links are represented as the Euclidean distance between the Cα of the cross-linked residues, and indicated in red (if below DSSO maximal distance restraint) or in magenta (if exceeding DSSO maximal distance restraint).

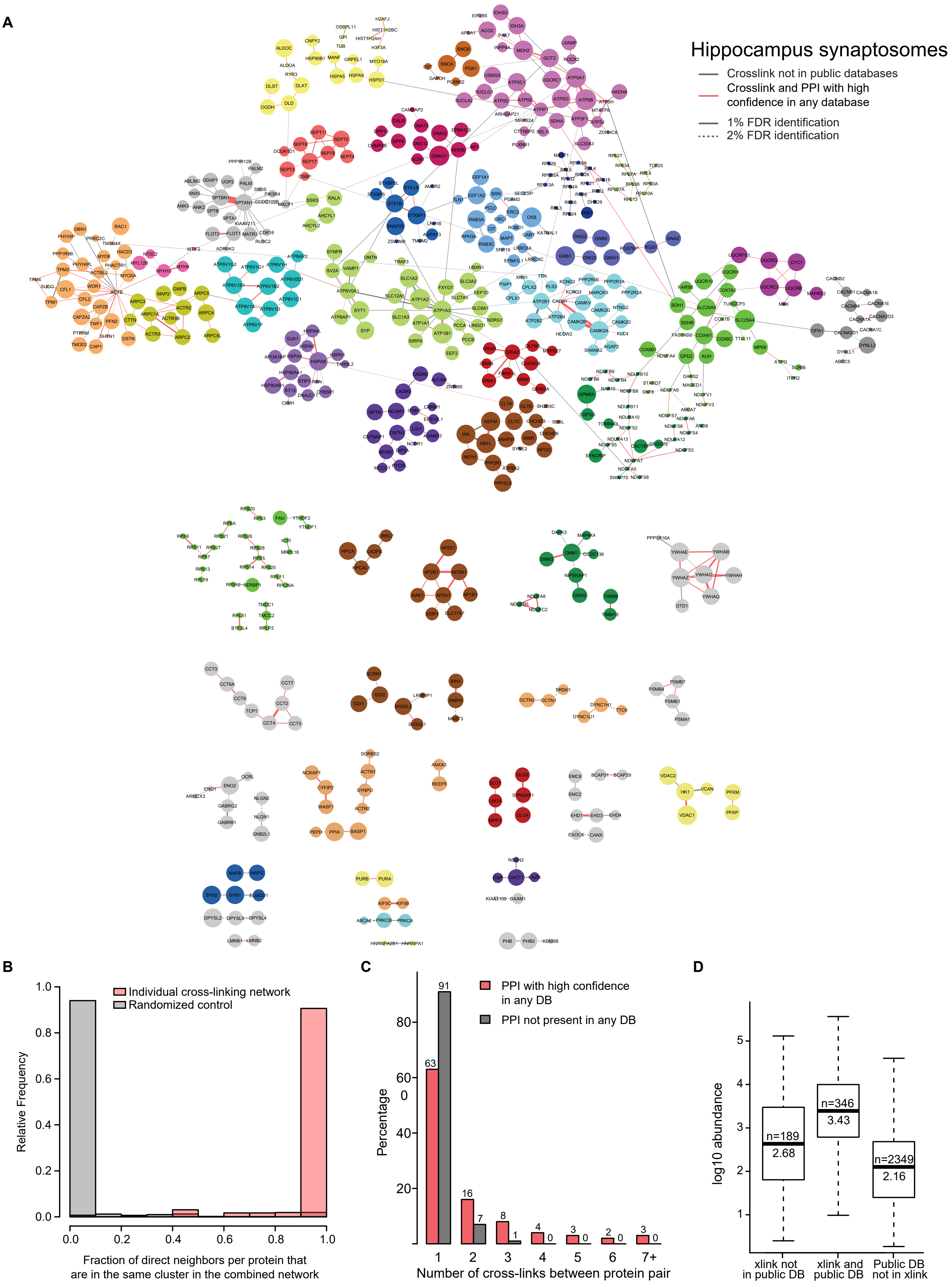
A
Hippocampus synaptosomes
Crosslink not in public databases
Crosslink and PPI with high confidence in any database
1% FDR identification
2% FDR identification
B
Individual cross-linking network
Randomized control
Relative Frequency
1.0
0.8
0.6
0.4
0.2
0.0
0.0
0.2
0.4
0.6
0.8
1.0
Fraction of direct neighbors per protein that are in the same cluster in the combined network
C
PPI with high confidence in any DB
PPI not present in any DB
Percentage
80
60
0
40
20
91
63
16
7
8
1
4
0
3
0
2
0
3
0
1
2
3
4
5
6
7+
Number of cross-links between protein pair
D
log10 abundance
5
4
3
2
1
n=189
2.68
n=346
3.43
n=2349
2.16
xlink not in public DB
xlink and public DB
Public DB not in xlink

Fig. S3. Characterization of XL-based protein interaction network from hippocampus synaptosomes. (**A**) Nodes represent proteins and edges show the interactions identified by a single XL-MS experiment in hippocampal synaptosomes (dataset 1). Protein names are included and disconnected protein modules with at least 3 proteins connected are shown. Each node represents a protein and the edge the PPI identified by cross-linking. Nodes are color-coded based on functional clusters shown in the combined network (Fig. 3A). Edges in red indicate protein-protein interactions (PPIs) found in both XL-MS data and in literature (high confidence in at least one database of STRING, InWEB and BioGRID). Sizes of the nodes are proportional to the protein abundances (Log2 scale). Widths of the edges are proportional to the number of unique Lys-Lys cross-links. The confidence of each cross-link is indicated as dotted and solid lines for 2% FDR and 1% FDR, respectively. All information regarding individual PPIs can be retrieved at http://xlink.cncr.nl. (**B**) Distribution of the fraction of direct neighbors (cross-linked proteins pairs) that are in the same cluster in the combined network (labelled in orange) and a randomly rewired network (100 iterations, labelled in light blue). (**C**) Distribution of the number of cross-links found between protein pairs present in at least one database (DB) with high confidence (labelled in red) or do not present in any of the three databases (i.e. STRING, InWEB and BioGRID, labelled in black). (**D**) Boxplot showing the distribution of protein abundances in different categories: xlink, cross-linked proteins identified in this study; public DB, protein interactions present in at least one public database with high confidence (i.e. STRING, InWEB and BioGRID). The number of proteins and median abundance are indicated in each box. Protein abundance data was obtained from (*12*).

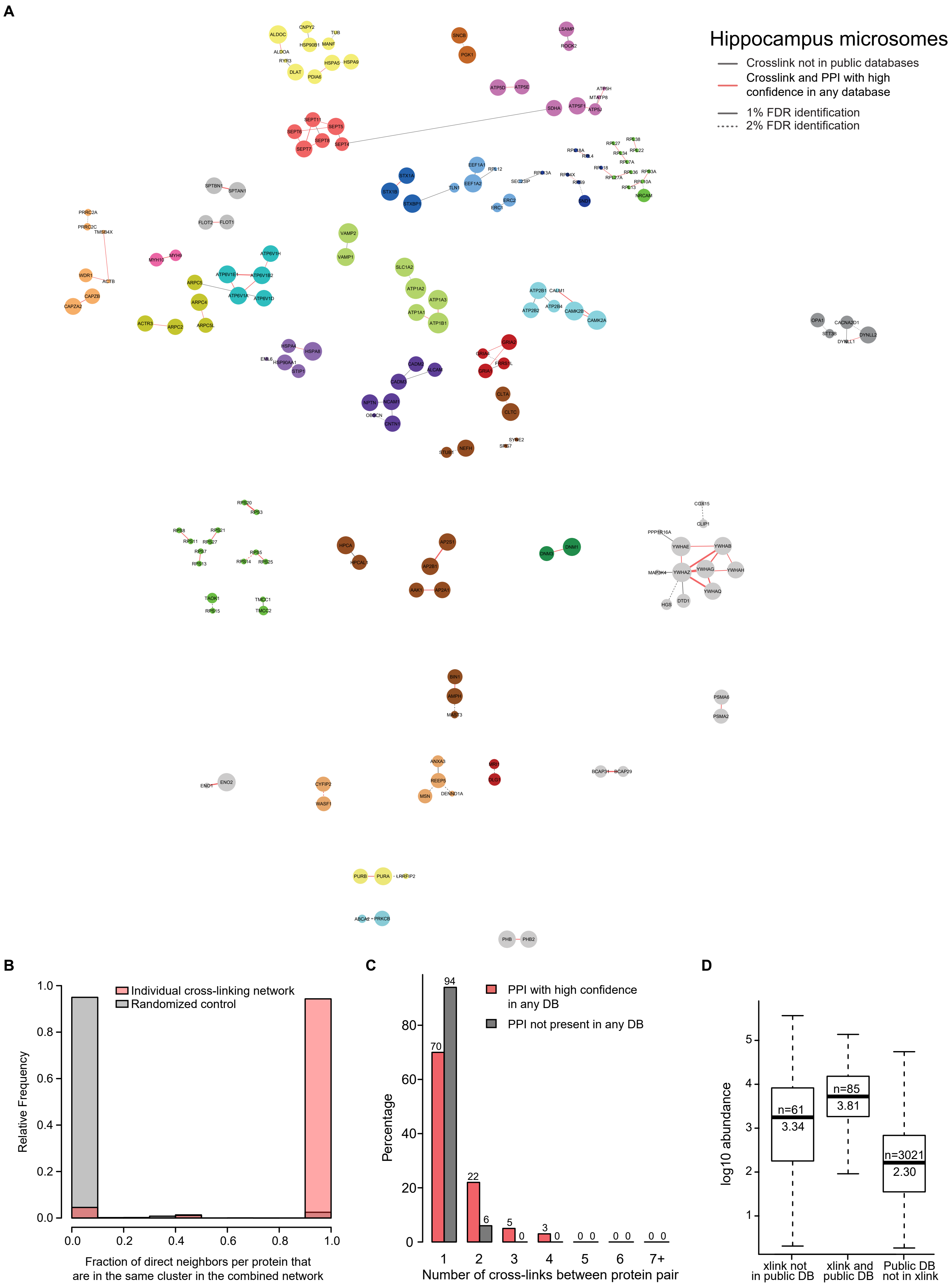

A
Hippocampus microsomes
Crosslink not in public databases
Crosslink and PPI with high confidence in any database
1% FDR identification
2% FDR identification
ALDOC
CNPY2
TUB
HSP90B1
MANF
ALDOA
RYR3
HSPA5
HSPA9
DLAT
PDIA6
SNCB
PGK1
LSAMP
ROCK2
ATP5D
ATP5E
SDHA
ATP5F1
ATP5J
ATP5H
MTATP8
SEPT11
SEPT5
SEPT6
SEPT8
SEPT7
SEPT4
STX1A
STX1B
STXBP1
TLN1
EEF1A1
EEF1A2
ERC2
ERC1
NRCAM
SPTBN1
SPTAN1
FLOT2
FLOT1
PRRC2A
PRRC2C
TMSB4X
ACTB
WDR1
CAPZB
CAPZA2
MYH10
MYH9
ARPC5
ARPC4
ACTR3
ARPC2
ARPC5L
ATP6V1H
ATP6V1E1
ATP6V1B2
ATP6V1A
ATP6V1D
VAMP2
VAMP1
SLC1A2
ATP1A2
ATP1A3
ATP1A1
ATP1B1
ATP2B1
CALM1
ATP2B2
ATP2B4
CAMK2B
CAMK2A
OPA1
CACNA2D1
STT3B
DYNLL2
DYNLL1
HSPA4
HSPA8
HSP90AA1
STIP1
CADM2
ALCAM
CADM1
NPTN
NCAM1
CNTN1
GRIA2
GRIA4
GRIA1
CLTA
CLTC
NEFH
STUB1
RPS20
RPS3
RPS8
RPS21
RPS11
RPS27
RPS7
RPS5
RPS13
RPS14
RPS25
TAOK1
RPS15
TMCC1
TMCC2
HPCA
HPCAL1
AP2S1
AP2B1
AAK1
AP2A1
DNM3
DNM1
COX15
CLIP1
PPP1R16A
YWHAE
YWHAB
MAP3K4
YWHAZ
YWHAG
YWHAH
YWHAQ
HGS
DTD1
BIN1
AMPH
MAST3
PSMA6
PSMA2
ANXA3
REEP5
MSN
DENND1A
MRI1
DLG1
BCAP31
BCAP29
ENO1
ENO2
CYFIP2
WASF1
PURB
PURA
LRRFIP2
ABCA2
PRKCB
PHB
PHB2
B
Individual cross-linking network
Randomized control
Relative Frequency
Fraction of direct neighbors per protein that are in the same cluster in the combined network
1.0
0.8
0.6
0.4
0.2
0.0
C
PPI with high confidence in any DB
PPI not present in any DB
Percentage
Number of cross-links between protein pair
94
70
22
6
5
0
3
0
0 0
0 0
0 0
1
2
3
4
5
6
7+
D
log10 abundance
n=61
3.34
n=85
3.81
n=3021
2.30
xlink not in public DB
xlink and public DB
Public DB not in xlink

Fig. S4. Characterization of XL-based protein interaction network from hippocampus microsomes. (**A**) Nodes represent proteins and edges show the interactions identified by a single XL-MS experiment in hippocampal microsomes (dataset 1). Protein names are included and disconnected protein modules with at least 3 proteins connected are shown. Each node represents a protein and the edge the PPI identified by cross-linking. Nodes are color-coded based on functional clusters shown in the combined network (Fig. 3A). Edges in red indicate protein-protein interactions (PPIs) found in both XL-MS data and in literature (high confidence in at least one database of STRING, InWEB and BioGRID). Sizes of the nodes are proportional to the protein abundances (Log2 scale). Widths of the edges are proportional to the number of unique Lys-Lys cross-links. The confidence of each cross-link is indicated as dotted and solid lines for 2% FDR and 1% FDR, respectively. All information regarding individual PPIs can be retrieved at http://xlink.cncr.nl. (**B**) Distribution of the fraction of direct neighbors (cross-linked proteins pairs) that are in the same cluster in the combined network (labelled in orange) and a randomly rewired network (100 iterations, labelled in light blue). (**C**) Distribution of the number of cross-links found between protein pairs present in at least one database (DB) with high confidence (labelled in red) or do not present in any of the three databases (i.e. STRING, InWEB and BioGRID, labelled in black). (**D**) Boxplot showing the distribution of protein abundances in different categories: xlink, cross-linked proteins identified in this study; public DB, protein interactions present in at least one public database with high confidence (i.e. STRING, InWEB and BioGRID). The number of proteins and median abundance are indicated in each box. Protein abundance data was obtained from (*12*).

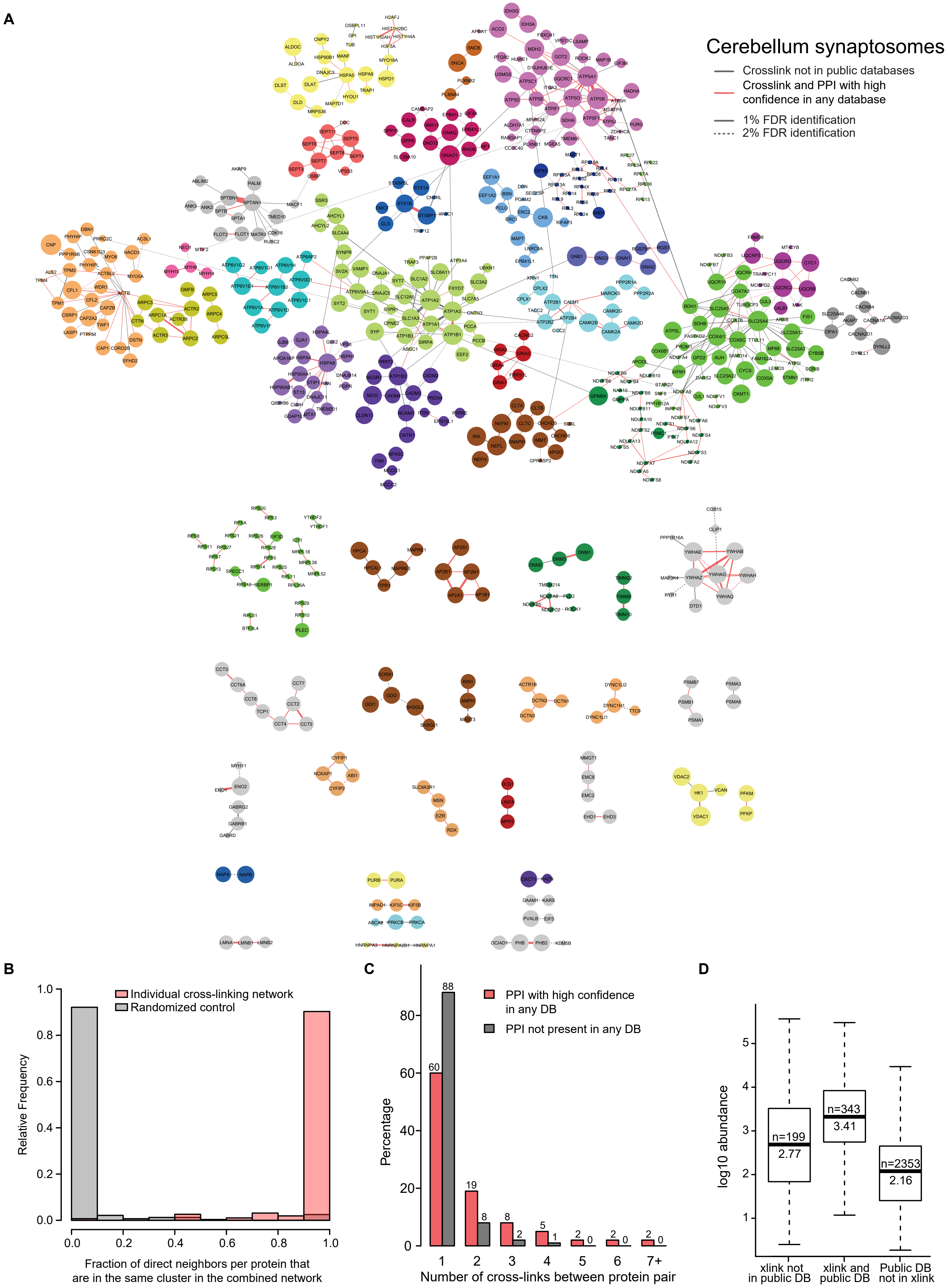
A
Cerebellum synaptosomes
Crosslink not in public databases
Crosslink and PPI with high confidence in any database
1% FDR identification
2% FDR identification
B
Individual cross-linking network
Randomized control
Relative Frequency
1.0
0.8
0.6
0.4
0.2
0.0
0.0
0.2
0.4
0.6
0.8
1.0
Fraction of direct neighbors per protein that are in the same cluster in the combined network
C
PPI with high confidence in any DB
PPI not present in any DB
Percentage
0
20
40
60
80
60
88
19
8
8
2
5
1
2
0
2
0
2
0
1
2
3
4
5
6
7+
Number of cross-links between protein pair
D
log10 abundance
1
2
3
4
5
n=199
2.77
n=343
3.41
n=2353
2.16
xlink not in public DB
xlink and public DB
Public DB not in xlink

Fig. S5. Characterization of XL-based protein interaction network from cerebellum synaptosomes. (**A**) Nodes represent proteins and edges show the interactions identified by a single XL-MS experiment in cerebellar synaptosomes (dataset 1). Protein names are included and disconnected protein modules with at least 3 proteins connected are shown. Each node represents a protein and the edge the PPI identified by cross-linking. Nodes are color-coded based on functional clusters shown in the combined network (Fig. 3A). Edges in red indicate protein-protein interactions (PPIs) found in both XL-MS data and in literature (high confidence in at least one database of STRING, InWEB and BioGRID). Sizes of the nodes are proportional to the protein abundances (Log2 scale). Widths of the edges are proportional to the number of unique Lys-Lys cross-links. The confidence of each cross-link is indicated as dotted and solid lines for 2% FDR and 1% FDR, respectively. All information regarding individual PPIs can be retrieved at http://xlink.cncr.nl. (**B**) Distribution of the fraction of direct neighbors (cross-linked proteins pairs) that are in the same cluster in the combined network (labelled in orange) and a randomly rewired network (100 iterations, labelled in light blue). (**C**) Distribution of the number of cross-links found between protein pairs present in at least one database (DB) with high confidence (labelled in red) or do not present in any of the three databases (i.e. STRING, InWEB and BioGRID, labelled in black). (**D**) Boxplot showing the distribution of protein abundances in different categories: xlink, cross-linked proteins identified in this study; public DB, protein interactions present in at least one public database with high confidence (i.e. STRING, InWEB and BioGRID). The number of proteins and median abundance are indicated in each box. Protein abundance data was obtained from (*12*).

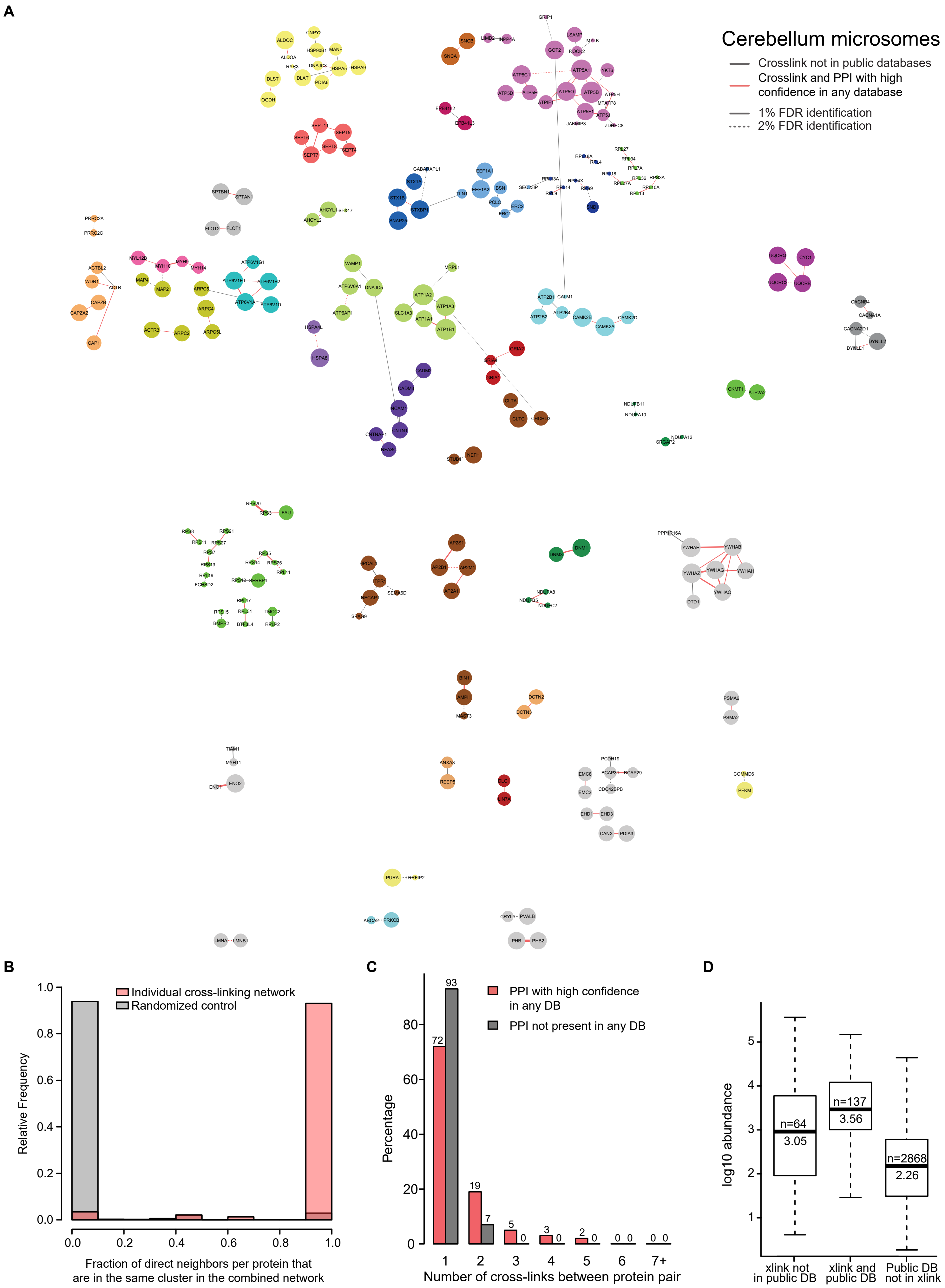
A
Cerebellum microsomes
Crosslink not in public databases
Crosslink and PPI with high confidence in any database
1% FDR identification
2% FDR identification
B
Individual cross-linking network
Randomized control
Relative Frequency
1.0
0.8
0.6
0.4
0.2
0.0
0.0 0.2 0.4 0.6 0.8 1.0
Fraction of direct neighbors per protein that are in the same cluster in the combined network
C
PPI with high confidence in any DB
PPI not present in any DB
Percentage
0 20 40 60 80
72 93
19 7
5 0
3 0
2 0
0 0
0 0
1 2 3 4 5 6 7+
Number of cross-links between protein pair
D
log10 abundance
1 2 3 4 5
n=64
3.05
n=137
3.56
n=2868
2.26
xlink not in public DB
xlink and public DB
Public DB not in xlink

Fig. S6. Characterization of XL-based protein interaction network from cerebellum microsomes. (**A**) Nodes represent proteins and edges show the interactions identified by a single XL-MS experiment in cerebellar microsomes (dataset 1). Protein names are included and disconnected protein modules with at least 3 proteins connected are shown. Each node represents a protein and the edge the PPI identified by cross-linking. Nodes are color-coded based on functional clusters shown in the combined network (Fig. 3A). Edges in red indicate protein-protein interactions (PPIs) found in both XL-MS data and in literature (high confidence in at least one database of STRING, InWEB and BioGRID). Sizes of the nodes are proportional to the protein abundances (Log2 scale). Widths of the edges are proportional to the number of unique Lys-Lys cross-links. The confidence of each cross-link is indicated as dotted and solid lines for 2% FDR and 1% FDR, respectively. All information regarding individual PPIs can be retrieved at http://xlink.cncr.nl. (**B**) Distribution of the fraction of direct neighbors (cross-linked proteins pairs) that are in the same cluster in the combined network (labelled in orange) and a randomly rewired network (100 iterations, labelled in light blue). (**C**) Distribution of the number of cross-links found between protein pairs present in at least one database (DB) with high confidence (labelled in red) or do not present in any of the three databases (i.e. STRING, InWEB and BioGRID, labelled in black). (**D**) Boxplot showing the distribution of protein abundances in different categories: xlink, cross-linked proteins identified in this study; public DB, protein interactions present in at least one public database with high confidence (i.e. STRING, InWEB and BioGRID). The number of proteins and median abundance are indicated in each box. Protein abundance data was obtained from (*12*).

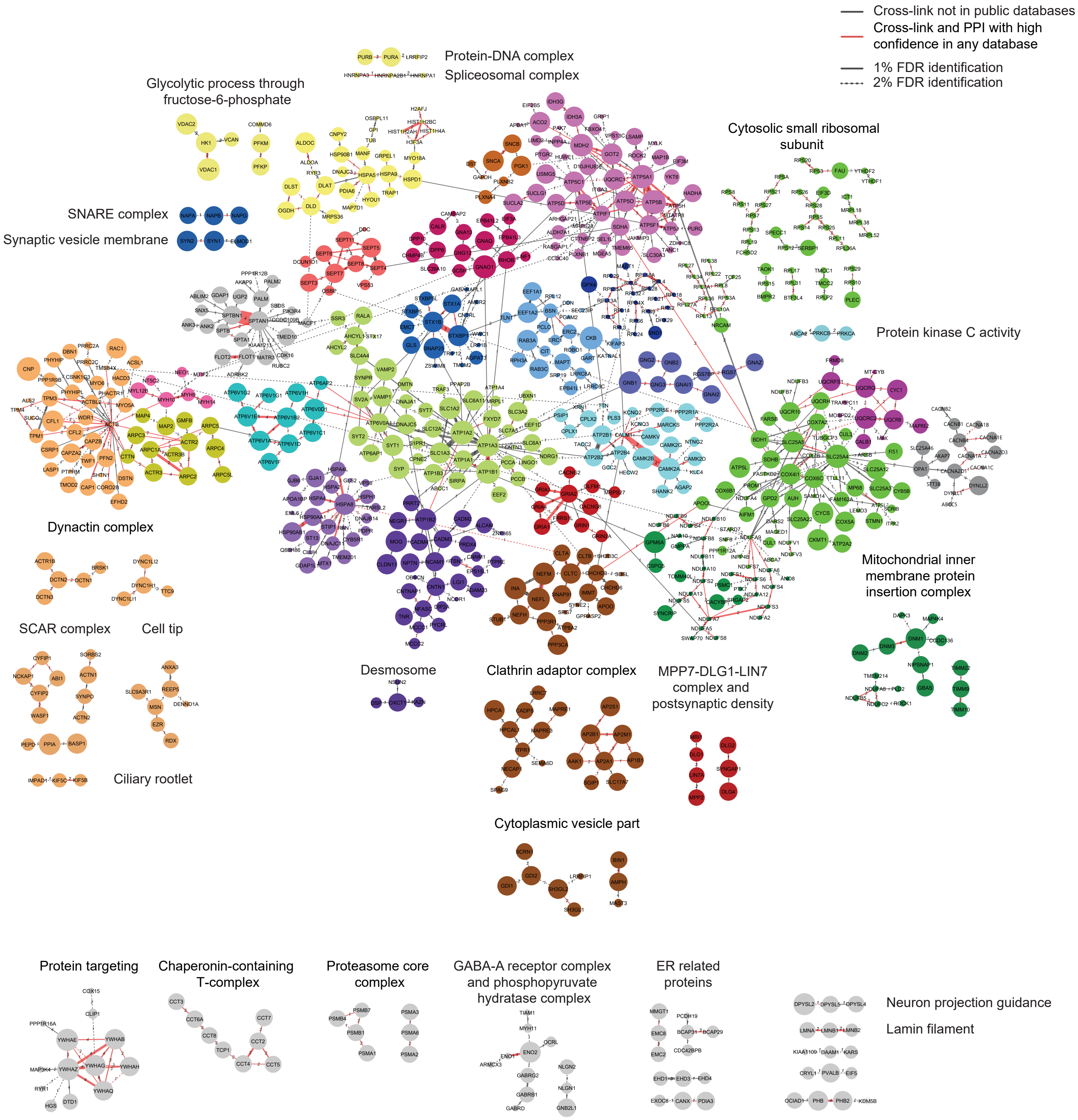
Cross-link not in public databases
Cross-link and PPI with high confidence in any database
1% FDR identification
2% FDR identification
Protein-DNA complex
Spliceosomal complex
Glycolytic process through fructose-6-phosphate
Cytosolic small ribosomal subunit
SNARE complex
Synaptic vesicle membrane
Protein kinase C activity
Dynactin complex
SCAR complex
Cell tip
Ciliary rootlet
Desmosome
Clathrin adaptor complex
MPP7-DLG1-LIN7 complex and postsynaptic density
Mitochondrial inner membrane protein insertion complex
Cytoplasmic vesicle part
Protein targeting
Chaperonin-containing T-complex
Proteasome core complex
GABA-A receptor complex and phosphopyruvate hydratase complex
ER related proteins
Neuron projection guidance
Lamin filament

Fig. S7. Extended and detailed XL-based protein interaction network (extended Fig. 3A). Nodes represent proteins and edges show the interactions identified by XL-MS (dataset 1). Protein names are included and disconnected protein modules with at least 3 proteins connected are shown. Each node represents a protein and the edge the PPI identified by cross-linking. Nodes are color-coded based on functional clusters generated by unsupervised edge-betweenness clustering for the core component or gene functional classification for the disconnected modules, and annotated by GO enrichment analysis. Disconnected modules were located next to the functionally closest cluster of the core component of the network. Edges in red indicate protein-protein interactions (PPIs) found in both XL-MS data and in literature (high confidence in at least one database of STRING, InWEB and BioGRID). Sizes of the nodes are proportional to the protein abundances (Log2 scale). Widths of the edges are proportional to the number of unique Lys-Lys cross-links. The confidence of each cross-link is indicated as dotted and solid lines for 2% FDR and 1% FDR, respectively. Edges are labeled according to the number of samples in which the PPI was identified. All information regarding individual PPIs can be retrieved at http://xlink.cncr.nl.

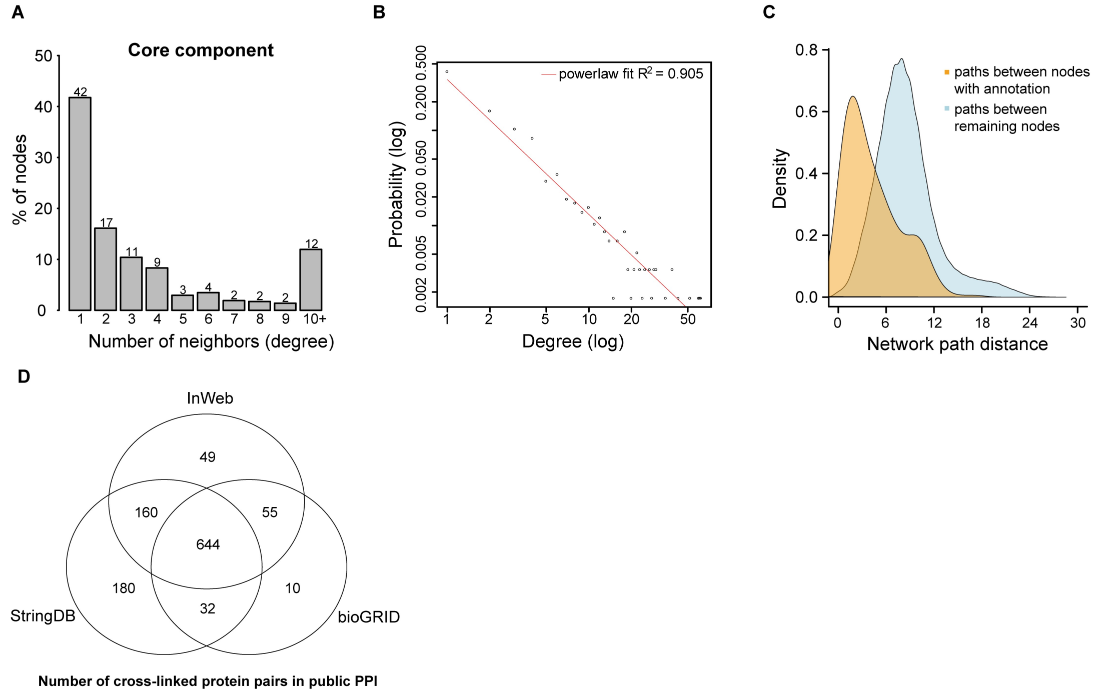

Fig. S8. XL-based protein interaction network analysis. (**A**) Degree distribution of the XL-based protein interaction network (Fig. 3A). (**B**) Power law log-log plot of the network. X-axis represents the degree of a node (number of edges) and y-axis represents the probability that a node with a given degree exists in the network. (**C**) Density plot of the path lengths between the proteins annotated with the same GO term and between all non−annotated nodes in the core component of the network. (**D**) Number of cross-linked protein pairs found in at least one of the public PPI databases (i.e., StringDB, InWEB and BrioGRID) with any confidence level.

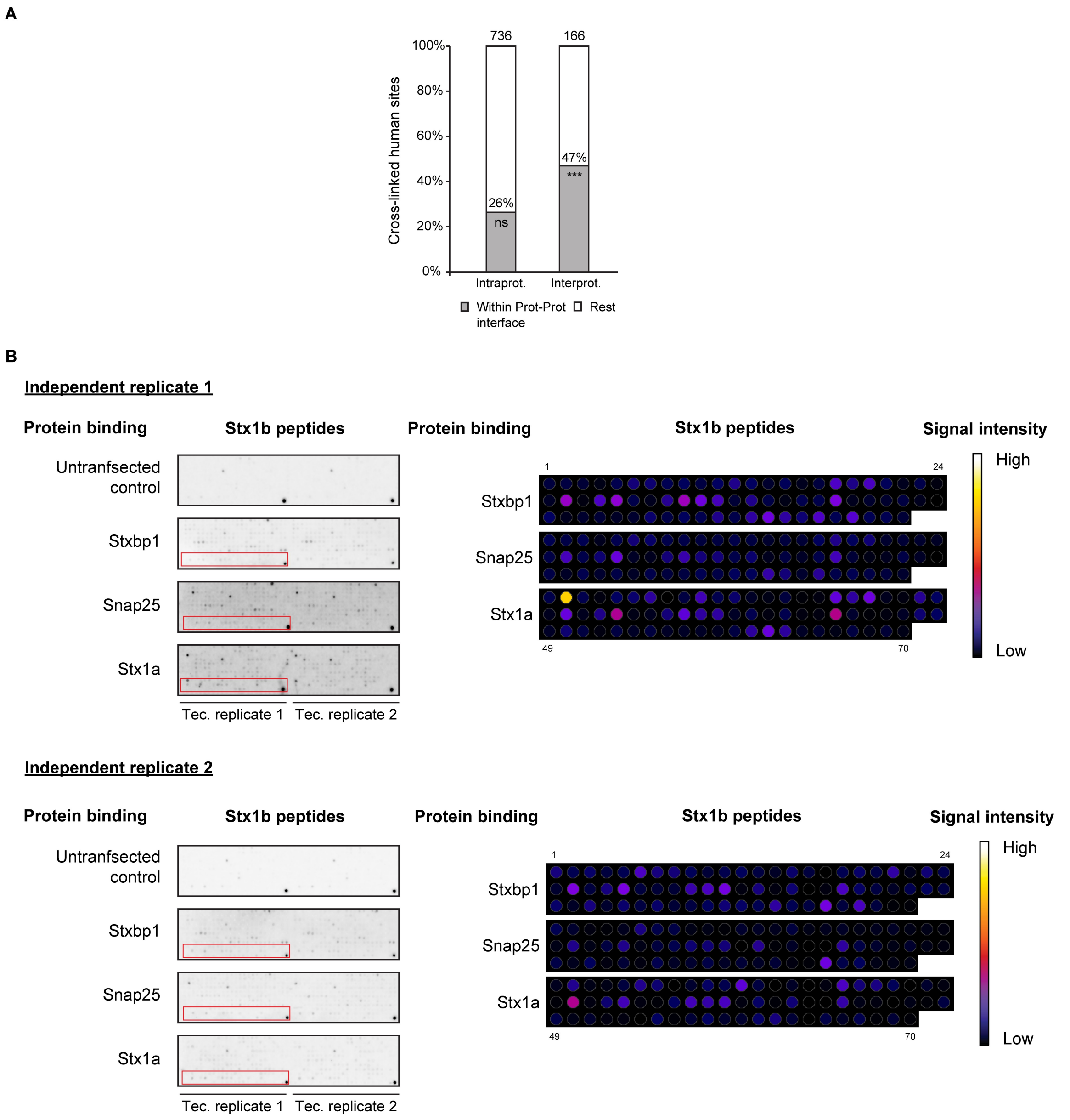

Fig. S9. Protein interaction interfaces and peptide array analysis (extended Fig. 4). (**A**) Percentage and total number of cross-linked lysines within and beyond the protein-protein interaction interfaces predicted by InteractomeINSIDER for the human proteome (Table S3). Fisher exact test showed a significant enrichment for interprotein cross-links within the interaction interfaces (*** <0.00001), but not for intraprotein cross-links (ns, 0.79). (**B**) The signal intensity detected by immunoblotting for each peptide spot (red squares) was processed by Protein Array Analyzer of ImageJ. Two independent replicates and two technical replicates were performed for each protein tested. Peptide sequences are presented in Table S4.

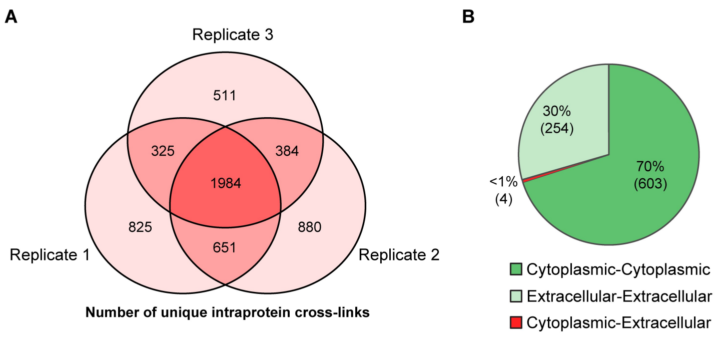

Fig. S10. Evaluation of XL-MS approach on biologically independent replicates for hippocampal synaptosome (extended Fig. 6). (**A**) Venn diagram showing the number of identified intraprotein cross-links over the different replicates. (**B**) Pie chart showing the number of cross-links identified between cytoplasmic and extra-cellular regions of proteins (dataset 2).

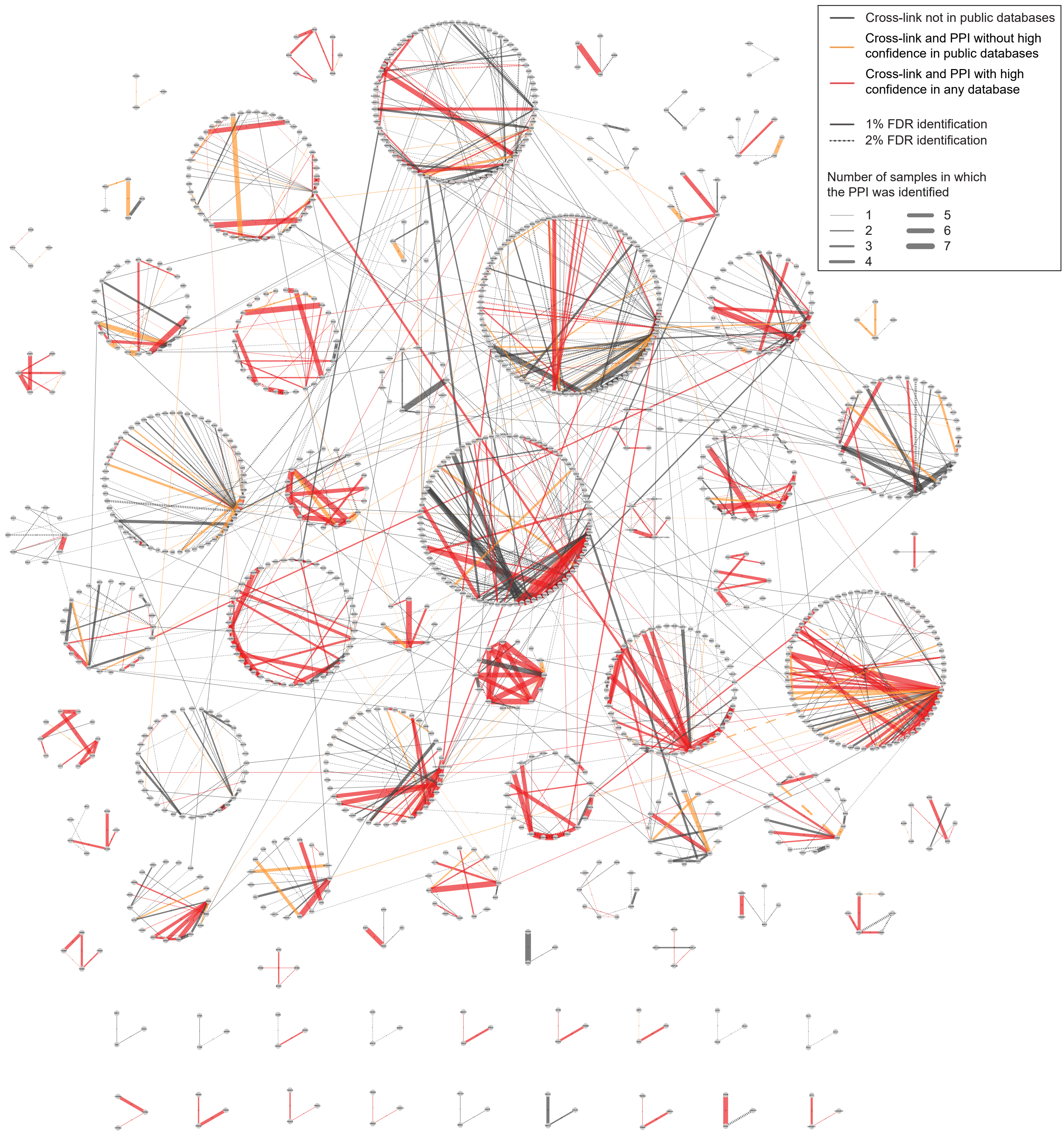
Cross-link not in public databases
Cross-link and PPI without high confidence in public databases
Cross-link and PPI with high confidence in any database
1% FDR identification
2% FDR identification
Number of samples in which the PPI was identified
1
2
3
4
5
6
7

Fig. S11. Complete XL-based protein interaction network from all seven cross-linking MS experiments. Nodes represent proteins and edges show the interactions identified by XL-MS (dataset 1 and 2). Disconnected protein modules with at least 3 proteins connected are shown. Nodes layout is based on unsupervised edge-betweenness clustering. Edges in red and orange indicate protein-protein interactions (PPIs) reported in literature (STRING, InWEB and BioGRID) classified with and without high confidence in at least one database, respectively. Labels and widths of the edges indicate the number of samples in which the PPI was identified. The confidence of each cross-link is indicated as dotted and solid lines for 2% FDR and 1% FDR, respectively. All information regarding individual PPIs can be retrieved at http://xlink.cncr.nl.
